# Understanding the toxicity induced by radiation-triggered neuroinflammation and the on-demand design of targeted peptide nanodrugs

**DOI:** 10.1038/s41392-025-02375-9

**Published:** 2025-09-04

**Authors:** Yue Shang, Xueyin Hu, Meixia Ren, Longbo Ma, Xiaoyu Zhao, Cong Gao, Lumeng Zhang, Shuqin Li, Luntao Liu, Bingwen Zou, Saijun Fan

**Affiliations:** 1 State Key Laboratory of Advanced Medical Materials and Devices, Tianjin Key Laboratory of Radiation Medicine and Molecular Nuclear Medicine, Tianjin Institutes of Health Science, Institute of Radiation Medicine, Chinese Academy of Medical Sciences & Peking Union Medical College, Tianjin, China; 2 Shengli Clinical Medical College of Fujian Medical University, Fujian Provincial Hospital, Fuzhou University Affiliated Provincial Hospital, Fuzhou, China; 3 Department of Surgery, Emory University School of Medicine, Atlanta, GA USA; 4 Division of Thoracic Tumor Multimodality Treatment and Department of Radiation Oncology, Cancer Center, West China Hospital, Sichuan University, Chengdu, China

**Keywords:** Neurological disorders, Drug development

## Abstract

Radiation-induced brain injury (RIBI) represents a severe complication of cranial radiotherapy, substantially diminishing patients’ quality of life. Unlike conventional brain injuries, RIBI evokes a unique chronic neuroinflammatory response that notably aggravates neurodegenerative processes. Despite significant progress in understanding the molecular mechanisms related to neuroinflammation, the specific and precise mechanisms that regulate neuroinflammation in RIBI and its associated toxicological effects remain largely unclear. Additionally, targeted neuroprotective strategies for RIBI are currently lacking. In this study, we systematically characterized the pathophysiology of RIBI using zebrafish (larvae/adults) and murine models. We established direct associations between neuronal damage and cognitive-behavioral deficits. Mechanistically, we proposed a ROS-mitochondrial-immune axis. Specifically, radiation-induced ROS lead to mitochondrial dysfunction, resulting in the leakage of mitochondrial DNA into the cytosol. This, in turn, activated the cGAS-STING pathway, thereby driving persistent microglia-mediated neuroinflammation. Furthermore, we engineered a dual-function nanotherapeutic agent, Pep-Cu_5.4_O@H151. This agent integrates ultrasmall copper-based nanozymes (Cu_5.4_O) for ROS scavenging and H151 (a STING inhibitor) and is conjugated with peptides that can penetrate the blood-brain barrier and target microglia. This nanoplatform exhibited excellent synergistic therapeutic efficacy by simultaneously neutralizing oxidative stress and blocking inflammatory cascades. Our research provided an in-depth analysis of radiation-induced neurotoxicity, clarifying the crucial ROS-mitochondrial-immune axis. Moreover, we have developed a precise therapeutic strategy on the basis of this mechanism.

## Introduction

Radiation-induced brain injury (RIBI) has emerged as a significant clinical challenge that undermines the long-term efficacy of radiotherapy.^1,2^ Epidemiological analyses have revealed that the cumulative incidence rates of RIBI in nasopharyngeal carcinoma survivors 4 years post-radiotherapy, in cohorts with poorly differentiated gliomas, and in patients undergoing stereotactic radiotherapy for meningiomas are alarmingly high. These incidences range from 1.9% to 5%, 1% to 24%, and 2.6% to 10%, respectively.^3–5^ RIBI has a unique radiation pathobiology that is distinct from that of conventional brain injuries, primarily due to the chronic neuroinflammatory response induced by radiation. This response significantly exacerbates neurodegenerative processes.^6–8^ Current therapeutic paradigms, which mainly involve high-dose glucocorticoid regimens combined with neurotrophic agents (such as gangliosides) and microcirculation modulators (such as beraprost sodium and benzopyran), provide only transient symptomatic relief and fail to offer a radical cure.^9–12^ Substantial progress has been made in elucidating the potential damage caused by RIBI. However, the toxicological effects of radiation-induced neuroinflammation at different developmental stages, as well as the underlying mechanisms, remain poorly understood. Moreover, targeted neuroprotective strategies to counteract the inevitable consequences of RIBI are still lacking.

Neuroinflammation is a cardinal pathological hallmark of central nervous system (CNS) disorders and serves as a critical driver of the pathogenesis and progression of various neurological conditions.^13–15^ The cyclic GMP–AMP synthase (cGAS)-stimulator of interferon genes (STING) signaling axis functions as a central regulatory hub within the innate immune response.^16–18^ Activating the cGAS-STING signaling pathway to increase the production of type I interferons (IFN-α/β) and proinflammatory cytokines has become a pivotal molecular strategy in oncotherapeutic approaches.^19,20^ However, compelling evidence indicates that excessive activation of the cGAS-STING signaling axis contributes to neuroinflammatory pathologies associated with various CNS disorders.^21–23^ For example, in Alzheimer’s disease (AD), amyloid-β (Aβ)-induced genomic DNA damage can trigger cGAS-STING hyperactivation, leading to microglial M1 polarization and subsequent proinflammatory cytokine storms.^24–26^ Similar pathological mechanisms have been observed in amyotrophic lateral sclerosis (ALS) and Parkinson’s disease (PD). Specifically, the accumulation of pathogenic proteins such as misfolded SOD1 and α-synuclein, along with mitochondrial dysfunction, results in the leakage of mitochondrial DNA (mtDNA) into the cytosol. This leakage activates the NLRP3 inflammasome via cGAS-STING-dependent pathways.^27,28^

Although emerging evidence has highlighted the involvement of cGAS-STING signaling in neuroinflammatory cascades across neurodegenerative and autoimmune disorders, the mechanistic role of this pathway in orchestrating radiation-induced neuropathology remains unclear. Mitochondria, which act as central hubs for ROS generation and ATP metabolism, exhibit increased radiosensitivity, mainly due to their structural vulnerabilities associated with circular double-stranded mtDNA.^29,30^ Consequently, radiation exposure can easily induce mitochondrial membrane potential collapse, leading to mtDNA leakage into the cytosol.^31^ cGAS can activate the cGAS-STING signaling axis in response to viral or bacterial genomes, as can endogenous danger signals such as nuclear DNA leakage or abnormal mtDNA release. Therefore, we propose that the activation of the cGAS-STING signaling axis triggered by radiation-induced cytosolic mtDNA plays a crucial role in driving neuroinflammatory cascades during the pathogenesis of RIBI.

**Scheme 1:**
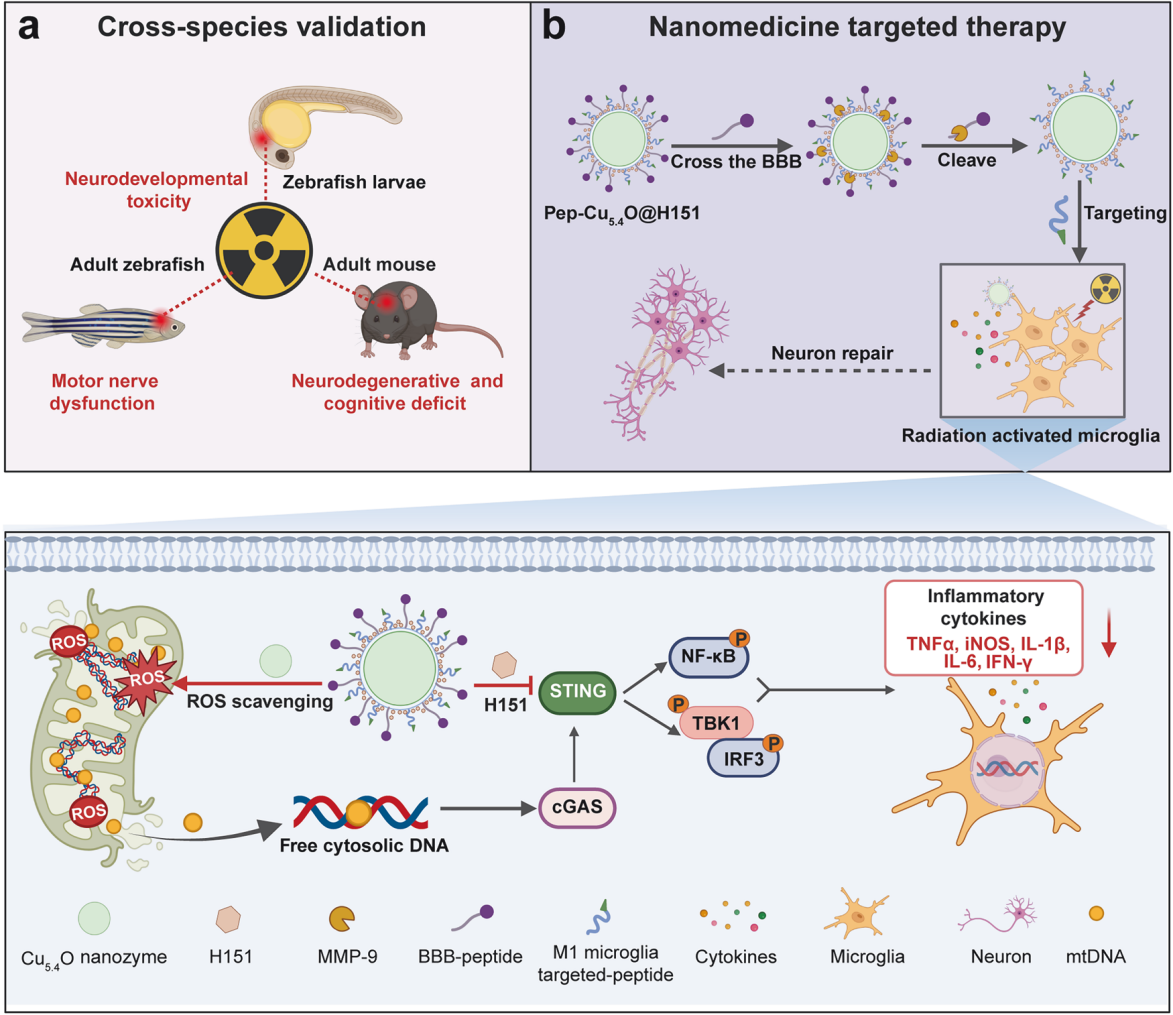
Schematic illustration of the investigation of the toxicological effects of radiation-induced neuroinflammation using cross-species models and engineered peptide nanodrugs for RIBI therapy. **a** Zebrafish (larvae/adults) and murine models were used to systematically examine the neurotoxic effects. **b** Subsequently, a novel peptide nanodrug system (Pep-Cu_5.4_O@H151) was developed. It integrates ultrasmall Cu_5.4_O nanozymes with broad-spectrum ROS scavenging capabilities and commercially available cGAS-STING axis inhibitors. This system, which includes BBB-penetrating peptides and microglial cell-targeting peptides, is designed to effectively cross the BBB and specifically target microglia for precise RIBI therapy. Mechanistically, mitochondrial dysfunction caused by radiation-induced ROS overproduction leads to cytosolic mtDNA leakage. This, in turn, excessively activates the cGAS-STING axis, resulting in a neuroinflammatory response. By leveraging the broad-spectrum ROS-scavenging properties of Cu_5.4_O nanozymes and the inhibitory effects of H151 on overactivation of the cGAS-STING axis, precise RIBI therapy can be achieved. Images were created using BioRender. Shang, Y. (2025) https://BioRender.com/s5 nlayu (agreement number: KJ28IJ4ZXF)

In this study, we systematically investigated the mechanisms of radiation-induced neuroinflammation and the corresponding toxicological effects in zebrafish larvae, adult zebrafish, and murine models (Scheme 1a). Additionally, we successfully developed a microglial cell-targeted nanodrug (Pep-Cu_5.4_O@H151). This nanodrug consists of ultrasmall Cu_5.4_O nanozymes, commercially available cGAS-STING axis inhibitors (H151), blood-brain barrier (BBB)-penetrating peptides, and microglial cell-targeted peptides, and it has been applied for precise RIBI therapy (Scheme 1b). Specifically, significant neurodevelopmental anomalies and apoptosis triggered by radiation were observed in both larval and adult zebrafish. These changes were accompanied by notable photoresponse deficits and locomotor dysfunction. To further investigated radiation-induced chronic neurotoxicity, murine models of RIBI were constructed by simulating clinical radiotherapy with a fractionated dose of 25 Gy. A 4-month longitudinal study revealed that radiation exposure triggered persistent neuroinflammation, leading to progressive CNS damage and potentially increasing the susceptibility of the subjects to neurodegenerative processes. Mechanistically, mitochondrial dysfunction caused by radiation-generated overexproduction ROS drives cytosolic mtDNA leakage. This leakage, in turn, excessively activates the cGAS-STING axis, ultimately resulting in a neuroinflammatory response. By leveraging the broad-spectrum ROS-scavenging properties of Cu_5.4_O nanozymes, the inhibitory effects of H151 on the overactivation of the cGAS-STING axis, and the BBB-penetrating and microglial cell-targeted properties of the peptides, precise RIBI therapy was achieved through the administration of Pep-Cu_5.4_O@H151 nanodrugs. Consequently, a treatment strategy combining “endogenous pathway inhibition and exogenous microenvironment modulation” has been proposed to alleviate radiation-induced neuroinflammation, thereby enhancing the efficacy of RIBI therapy. This work elucidates the role of the mitochondrial-immune axis in RIBI and opens new avenues for the precise treatment of neuroinflammatory-related diseases.

## Results

### Radiation-induced neurodevelopmental deficits in zebrafish larvae

The zebrafish embryo has emerged as a crucial vertebrate model for investigating neural developmental injuries and repair mechanisms. This is due to its remarkable biological characteristics, including over 80% homology in neurodevelopment-related genes with those of humans.^32^ Its ability to produce optically transparent larvae allows direct visualization of dynamic neurodevelopmental processes such as neural progenitor proliferation, differentiation, and migration.^33,34^ This provides empirical insights into spatiotemporal cellular behaviors.

**Fig. 1:**
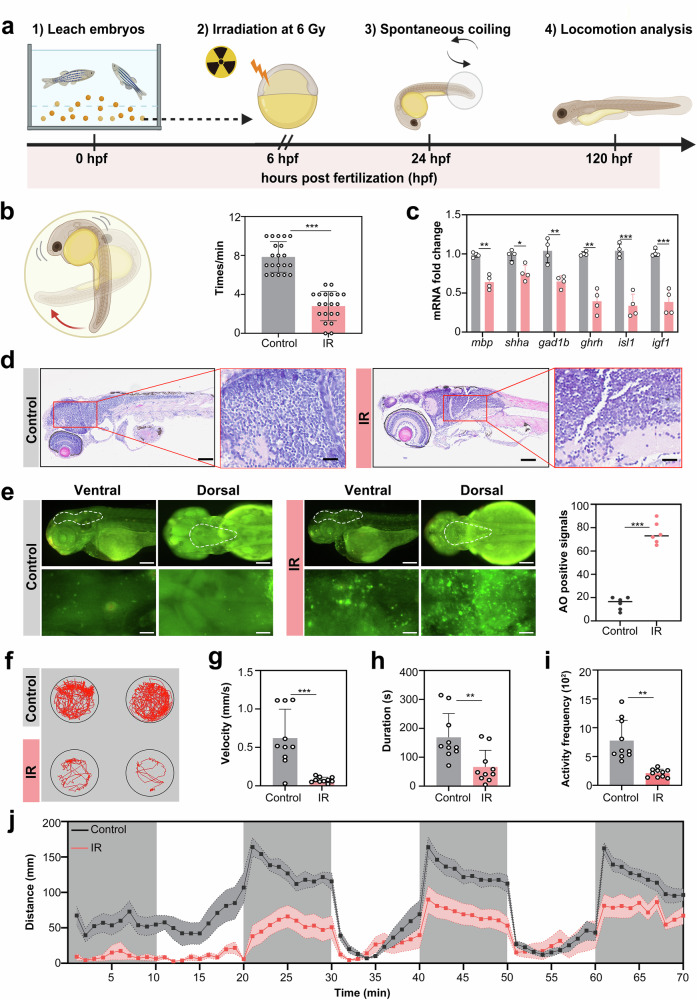
Radiation-induced neurodevelopmental deficits in zebrafish larvae. **a** Experimental timeline for radiation exposure and behavioral assessment. Embryos at 6 hpf were irradiated with 6 Gy γ rays (0.84 Gy/min, 7 min). Spontaneous coiling behavior was analyzed at 24 hpf, followed by locomotion analysis at 120 hpf. **b** Schematic illustration of spontaneous embryonic coiling patterns (a coiling event: tail bending ≥ 90°). Coiling frequency was quantified within 1-minute intervals (*n* = 20 embryos per group; Student’s *t*-test). **c** mRNA expression levels of neurodevelopment-related genes (*mbp*, *shha*, *gad1b*, *ghrh*, *isl1*, *igf1*) at 120 hpf (*n* = 4 biological replicates; Student’s *t*-test). **d** Representative hematoxylin‒eosin (HE) staining images showing whole-body morphology (left) and brain histology (right, boxed region magnified). **e** Apoptosis analysis via acridine orange (AO) staining. Ventral/dorsal views of AO fluorescence, including whole-body morphology (top) and the brain area (bottom, the white dashed-line region magnified). The number of apoptotic cells in the brain was quantified (*n* = 6 larvae per group; Student’s *t*-test). **f–i** Locomotion behavior analysis at 120 hpf during 10 min of adaptation. swimming trajectories (**f**); velocity (**g**); movement duration (**h**); activity frequency (**i**). **j** Locomotor distance of zebrafish larvae under alternating light‒dark cycles. After 10 min of dark adaptation, the zebrafish were subjected to three consecutive cycles of photoperiod stimulation (10 min light/10 min dark). The gray shaded areas indicate dark phases; the white areas denote light phases (**f**–**j**: *n* = 10 larvae; Student’s *t* test). All the statistical data are presented as the means ± SEMs. Scale bars: 500 μm (**d**, left), 50 μm (**d**, right), 200 μm (**e**, top), and 50 μm (**e**, bottom)

However, our understanding of radiation-induced neurodevelopmental toxicity, especially early-onset molecular events that precede behavioral manifestations, is limited. In this study, we systematically designed a timeline using zebrafish larvae to dissect γ-ray-induced neural pathogenesis across different developmental stages (Fig. 1a). Notably, neuromuscular defects were observed as early as 24 hours post fertilization (hpf) after radiation exposure. There was a significant 42% reduction in embryonic coiling frequency at 24 hpf (Fig. 1b). These observations suggest impaired neuromuscular circuit formation, which is supported by the downregulation of key neurodevelopmental genes (*mbp*: myelination; *shha*: axon guidance; *gad1b*: GABAergic signaling) at 120 hpf (Fig. 1c). Histological examination revealed ventricular enlargement and disorganized optic tectum layering (Fig. 1d).

Radiation-triggered apoptosis was predominantly localized in the hindbrain regions, as shown by a significant increase in the green fluorescent signal in irradiated larvae (Fig. 1e). These findings highlight the radiosensitivity of brainstem nuclei, which may explain the vulnerability observed in clinical cases of radiation-induced brainstem necrosis. Compared with control larvae, irradiated zebrafish larvae presented a marked reduction in locomotor capacity, as indicated by decreased behavioral trajectory density (Fig. 1f). Quantitative behavioral tracking revealed that irradiated zebrafish larvae had decreased velocity (*p* < 0.001, Fig. 1g), reduced movement duration (*p* < 0.01, Fig. 1h), diminished activity frequency (*p* < 0.01, Fig. 1i), and attenuated light‒dark responsiveness (Fig. 1j). Notably, the sustained hypoactivity across photoperiod transitions suggests that radiation disrupts evolutionarily conserved arousal circuits, potentially through the dysregulation of Igf1/Ghrh signaling, which are key regulators of hypothalamic‒pituitary axis maturation.^35^ Collectively, these results clarify the temporal dynamics of radiation-induced neurodevelopmental toxicity during the embryonic period, thus enhancing our understanding of RIBI.

### Radiation-induced neuropathology in adult zebrafish brains

**Fig. 2:**
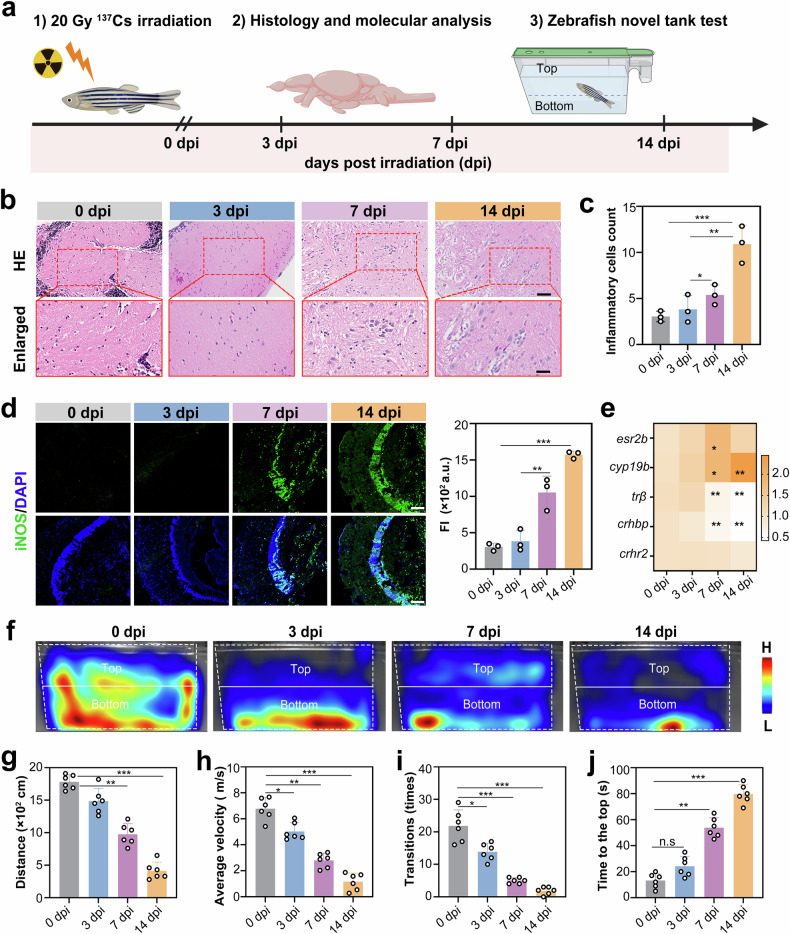
Radiation-induced neuropathology in adult zebrafish brains. **a** Experimental timeline for whole-body irradiation with 20 Gy γ-rays (0.84 Gy/min, 24 min) followed by subsequent analyses. Examinations were conducted at 3, 7, and 14 dpi. These included brain histology, molecular assays, and behavioral tests using novel tank locomotor analysis. **b** Representative HE-stained images of adult zebrafish brains. The boxed areas show enlarged views of the optic tectum. **c** Quantification of inflammatory infiltrates in the brain parenchyma (*n* = 3 brains per group; one-way ANOVA). **d** Representative images of iNOS immunofluorescence and quantification of fluorescence intensity (*n* = 3 brain sections; one-way ANOVA). **e** Heatmap of neuroendocrine-related gene mRNA expression (*esr2b*, *cyp19b*, *trβ*, *crhbp*, *crhr2*) across time points. **f–j** Novel tank behavioral profiling: heatmaps depicting spatial distribution (top/bottom zones demarcated by the horizontal midline) (**f**); total distance (**g**); average velocity (**h**); number of transitions (**i**); latency to enter the top (**j**) (**f**–**j**: *n* = 6 zebrafish; one-way ANOVA). All the statistical data are presented as the means ± SEMs. Scale bars: 200 μm (**b**, top), 50 μm (**b**, boxed areas), 100 μm (**d**)

To investigate the acute neurotoxic effects of radiation in mature vertebrates, we utilized adult zebrafish as a model organism. Adult zebrafish possess conserved neuroinflammatory and neuroendocrine pathways.^36,37^ Adult zebrafish were exposed to 20 Gy of whole-body irradiation. Subsequently, longitudinal assessments were conducted. These assessments included neurobehavioral profiling, histopathological examination, and quantification of molecular biomarkers at specified post-irradiation intervals (3, 7, and 14 days) to characterize the progression of radiation-induced cerebral lesions (Fig. 2a). Histopathological analysis revealed progressive structural degeneration in the optic tectum, a visual processing center that is homologous to the mammalian superior colliculus. This degeneration was characterized by disorganized neuronal layering (Fig. 2b). Radiation exposure triggered significant neuroinflammation. The number of inflammatory infiltrates increased 4.2-fold by 14 days post-irradiation (dpi) (*p* < 0.001, Fig. 2c). Additionally, there was a sustained induction of inducible nitric oxide synthase (iNOS). Compared with that of the control group, the fluorescence intensity of iNOS increased 5.3-fold (*p* < 0.001), indicating chronic oxidative stress-mediated neuronal damage (Fig. 2d).

Behavioral phenotyping revealed radiation-induced anxiety-like states in the zebrafish. This was evidenced by the reduced exploration of the top zone in the irradiated fish (Fig. 2f). Specifically, we observed a significant 68% decrease in the total motion distance (*p* < 0.001, Fig. 2g), a 72% reduction in the average velocity (*p* < 0.001, Fig. 2h), a decrease in the number of shuttling times from 20 to 3 (*p* < 0.001, Fig. 2i), and an increase in latency from 10.4 to 88 s (*p* < 0.001, Fig. 2j). These radiation-induced behavioral deficits in zebrafish are mechanistically linked to neuroendocrine dysregulation. This was evidenced by the significant downregulation of genes associated with the hypothalamic‒pituitary‒interrenal (HPI) axis at 7 dpi. For example, the expression level of corticotropin-releasing hormone binding protein (*crhbp*) decreased to 0.4-fold greater than that of the control, and the expression level of thyroid receptor *β* (*trβ*) decreased to 0.3-fold greater than that of the control. This transcriptional suppression was correlated with disrupted glucocorticoid/thyroid hormone signaling, suggesting that the compromised neuroprotective feedback mechanisms exacerbated radiation-induced neural circuit dysfunction. Importantly, the HPI axis is functionally analogous to the mammalian hypothalamic–pituitary–adrenal (HPA) axis.^38^ Additionally, the conserved upregulation of aromatase B (*cyp19b*), which is critical for neurosteroid synthesis, increased 2.1-fold. This upregulation highlights compromised neuroendocrine resilience and potentially exacerbates radiation-induced anxiety phenotypes (Fig. 2e). In conclusion, the zebrafish is a reliable translational model for studying radiation-associated neurotoxicity. The failure of endocrine‒immune crosstalk may underlie both the behavioral pathologies and histological neurodegeneration observed in this model.

### Focal χ-ray irradiation induces neuropathology in mouse brains

**Fig. 3:**
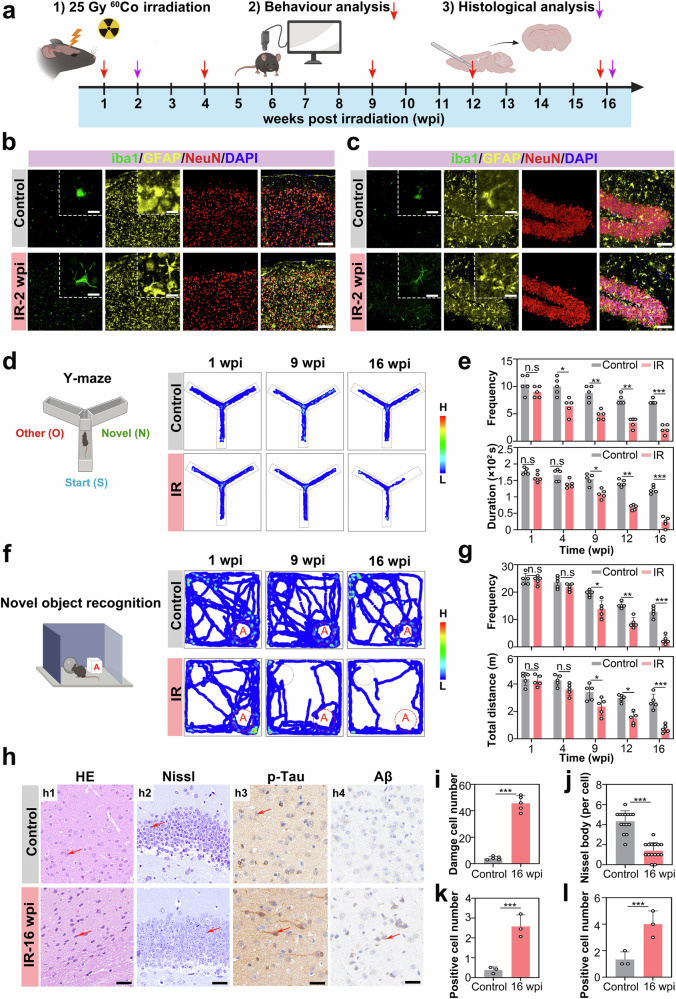
Focal χ-ray irradiation induces neuropathology in mouse brains. **a** Experimental timeline for focal-brain irradiation (25 Gy χ-rays) and subsequent analyses. Mice (6–8 weeks old) received a cumulative 25 Gy (5 Gy × 5 ^60^Co χ-ray focal brain irradiation, followed by behavioral tests at 1, 4, 9, 12, and 16 wpi, with histological analyses at 16 wpi. **b** and **c** Immunofluorescence characterization of activated glial morphology in the cortex (**b**) and hippocampus (**c**) of mice at 2 wpi. Green fluorescence: microglial marker iba1; yellow fluorescence: astrocyte marker GFAP; red fluorescence: neuronal marker NeuN. **d** Y-maze behavioral profiling. Schematic of Y maze compartments: start arm (S), other arm (O), and novel arm (N). Heatmaps of exploration patterns at 1, 9, and 16 wpi. **e** Y-maze cognitive metrics. Number of arm entries and durations in the novel arm (*n* = 5 mice; one-way ANOVA). **f** Novel object recognition test. Schematic showing novel object A (cube). Heatmaps of exploration at 1, 9, and 16 wpi. **g** Recognition performance. Interaction frequency and distance traveled in relation to the novel object (*n* = 5 mice; one-way ANOVA). **h** Histopathological analysis at 16 wpi. HE staining (**h1**), Nissl staining of the hippocampal CA1 region (**h2**), phosphorylated Tau (p-Tau) immunohistochemical (DAB) staining (**h3**) and phosphorylated amyloid-beta (p-Aβ) immunohistochemical (DAB) staining (**h4**) were performed. **i** Quantification of degenerating neurons (*n* = 5 mouse sections; Student’s *t* test). **j** Quantification of Nissl bodies (n = 15 cells; Student’s *t* test). **k** Quantification of p-Tau-positive signals (*n* = 3 mouse sections; Student’s *t* test). **l** Quantification of DAB signals (*n* = 3 mouse sections; Student’s *t* test). All the statistical data are presented as the means ± SEMs. Scale bars: 20 μm, 5 μm (**b**, **c**), 100 μm (**h1**), 50 μm (**h2**), 20 μm (**h3**), and 20 μm (**h4**)

We subsequently conducted an in-depth analysis of chronic radiation toxicity in mammalian cognitive circuits via a murine model of localized brain irradiation. Specifically, C57BL/6 mice (aged 6–8 weeks, corresponding to human early adulthood of 18–24 years) were subjected to focal cranial irradiation at a dose of 25 Gy. Longitudinal behavioral phenotyping was carried out over a 16-week observational period. Cognitive‒motor assessments performed at 1, 4, 9, 12, and 16 weeks post-irradiation (wpi), revealed a time-dependent functional decline (Fig. 3a).

The excessive activation of microglia and astrocytes constitutes a core mechanism driving the neuroinflammatory cascade.^39^ These two cell types sustain a vicious cycle in the neuroimmune microenvironment by coordinately releasing proinflammatory cytokines (e.g., TNF-α, IL-1β, and IL-6), ROS and neurotoxic mediators. At 2 wpi, we examined the activation status of two glial cell types in the cortex and hippocampus. Immunofluorescence staining revealed that microglia transitioned from a resting ramified morphology to an activated state, exhibiting hypertrophic morphology with pseudopodia-like processes. Moreover, astrocytes displayed features of reactive astrogliosis, characterized by somatic swelling and increased glial fibrillary acidic protein (GFAP) immunoreactivity (Fig. 3b, c). These findings suggest that ionizing radiation promotes microglial polarization toward a proinflammatory (M1) phenotype, whereas reactive astrogliosis may synergistically amplify neuroinflammatory cascades.

We evaluated the changes in the cognitive behaviors of the mice in a stage-specific manner. Y-maze behavioral profiling and novel object recognition (NOR) demonstrated progressive radiation-induced cognitive impairment. During the initial postirradiation phase (1 wpi), no significant differences were observed in novel arm exploration frequency (*p* = 0.214) or duration (*p* = 0.387). However, by 9 wpi, a marked cognitive decline emerged, characterized by a 43% reduction in novel arm entries (*p* < 0.01) and an 11% decrease in exploration time (*p* < 0.05). This deterioration culminated in severe spatial memory deficits at 16 wpi, with significant reductions in both the frequency (*p* < 0.001) and duration (*p* < 0.001) of novel arm exploration (Fig. 3d, e and Supplementary Fig. 1a). These findings suggest irreversible hippocampus-dependent memory consolidation impairment, which is consistent with human radiation-induced cognitive decline. In the NOR test, exploratory trajectories toward novel object A (cube) showed a significant reduction at 9 wpi, progressing to a virtual absence of exploration by 16 wpi (Fig. 3f and Supplementary Fig. 1b). Quantitative analysis revealed an exponential decay in the object interaction frequency, which decreased from 25.3 ± 2.1 events at 1 wpi to 3.2 ± 0.8 events at 16 wpi (*p* < 0.001). A concomitant decrease in movement distance (from 4.2 ± 0.9 m at 1 wpi to 0.9 ± 0.3 m at 16 wpi; *p* < 0.001) confirmed that this deficit reflected cognitive rather than motor impairment, suggesting selective hippocampal‒prefrontal circuit dysfunction (Fig. 3g). As a direct quantitative indicator of motor function, the overall decrease in movement distance directly reflects fundamental impairments in motor neural pathways caused by irradiation. However, in cognitive behavioral tests, its localized reduction in task-specific regions (e.g., novel arm and novel object exploration zone) essentially represents a secondary manifestation of cognitive deficits. The underlying mechanisms are as follows: spatial memory impairment can impair locomotor pathfinding efficiency, whereas executive function dysfunction may reduce sustained exploratory motivation, synergistically exacerbating the deterioration of motor phenotypes.

Histopathological evidence further revealed radiation-induced selective damage to the prefrontal‒hippocampal axis. HE staining revealed characteristic neurodegenerative alterations in the prefrontal cortex at 16 wpi, manifesting as pyknotic nuclei and neuronal vacuolization (Fig. 3h, i). The Nissl body density in the hippocampal CA1 region decreased to 37% of the control level (*p* < 0.001, Fig. 3h, j), indicating impaired synaptic plasticity due to radiation exposure. Quantitative neuropathological analysis revealed a 3.8-fold increase in phosphorylated Tau (p-Tau) positive neuronal density, which exhibited a strong inverse correlation with NOR performance (*p* < 0.001, Fig. 3h, k). Although conventional phosphorylated amyloid-beta (p-Aβ) plaques showed undetectable congophilic deposits, the emergence of faint cytoplasmic diaminobenzidine (DAB) positivity at 16 wpi was observed in neurons, suggesting that incipient Aβ oligomerization preceded plaque formation (*p* < 0.001, Fig. 3h, l). Terminal histopathological analysis at 16 wpi demonstrated that progressive hippocampal neurodegeneration was accompanied by chronic neuroinflammation. These multiscale findings confirmed that radiation potentially disrupts associative cortical networks critical for cognition through a tau-related pathogenic mechanism.

### Neuronal injury caused by radiation-induced microglial proinflammatory responses

**Fig. 4:**
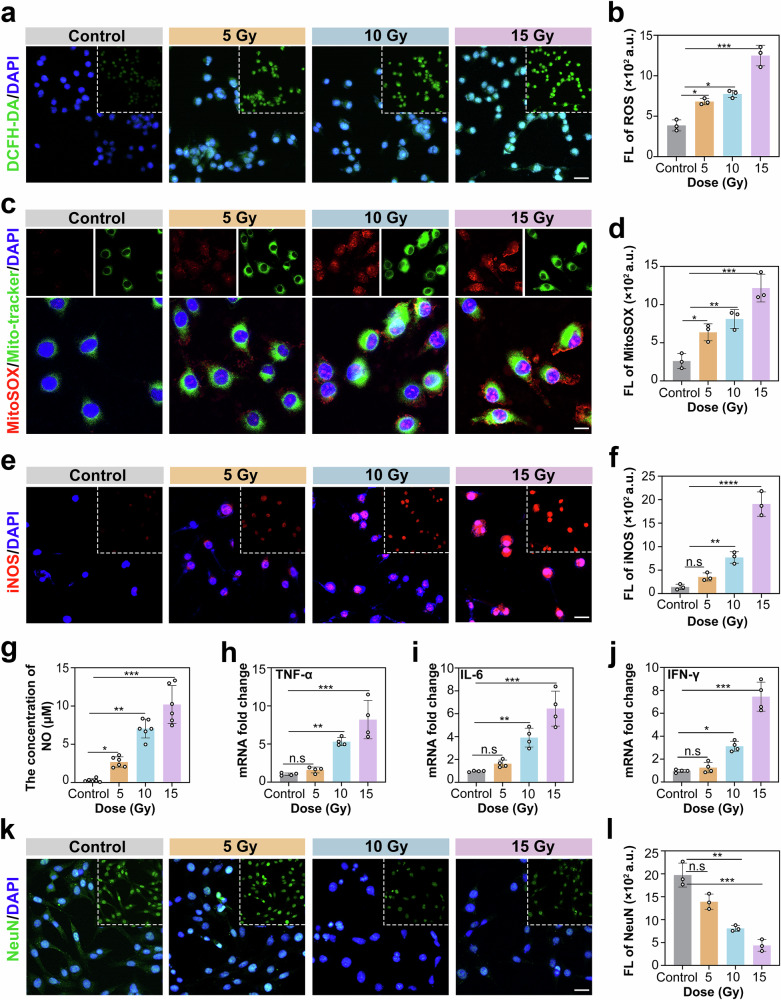
Neuronal injury caused by radiation-induced microglial proinflammatory responses. **a** Representative fluorescence micrographs of intracellular ROS detection via DCFH-DA staining in BV2 microglia at 6 hpi with 5, 10, and 15 Gy. **b** Quantification of ROS fluorescence intensity normalized to that of the control (*n* = 3 fields; one-way ANOVA). **c** Representative fluorescence micrographs of mtROS detection via MitoSOX staining in BV2 microglia at 6 hpi with 5, 10, and 15 Gy. **d** Quantification of MitoSOX fluorescence intensity normalized to that of the control (*n* = 3 fields; one-way ANOVA). **e** Immunofluorescence images of iNOS in BV2 cells at 24 hpi. **f** Quantification of iNOS fluorescence intensity relative to that of the control (*n* = 3 fields; one-way ANOVA). **g** Quantification of the NO concentration in the supernatant of BV2 cells (*n* = 6; one-way ANOVA). **h–j** Quantitative reverse transcription PCR analysis of the levels of the proinflammatory cytokines TNF-α (**h**), IL-6 (**i**), and IFN-γ (**j**) in irradiated BV2 cells (normalized to β-actin via the 2^−ΔΔCt^ method; *n* = 4 biological replicates; one-way ANOVA). **k** NeuN immunofluorescence micrographs of HT22 hippocampal neurons after 24 h of exposure to conditioned media from irradiated BV2 microglia. **l** Quantification of surviving neurons (NeuN^+^ cells/mm²), normalized to neurons treated with control conditioned media (*n* = 3; one-way ANOVA). All the statistical data are presented as the means ± SEMs. Scale bars: 20 μm (**a**, **c**, **e**, and **k**)

To explore the in vivo cognitive deficits and neurodegenerative pathology observed in irradiated mice, we mechanistically dissected microglia-mediated neuroinflammatory cascades via complementary in vitro cellular experiments. Radiation exposure elicited time- and dose-dependent microglial activation, as evidenced by an immediate surge of ROS in a murine microglial cell line (BV2). The fluorescence of 2',7'-dihydrofluorescein diacetate (DCFH-DA) increased 3.3-fold at a radiation dose of 15 Gy (*p* < 0.001, Fig. 4a, b). Ionizing radiation induces ROS through dual mechanisms. In addition to the direct radiolysis of cytoplasmic water molecules by radiation energy, mitochondrial electron transport chain damage causes electron leakage from complexes I/III, leading to increased production of mitochondrial ROS (mtROS).^40^ We employed the MitoSOX fluorescent probe in conjunction with a mitochondrial dye (MitoTracker) for co-staining, enabling the subcellular localization of mtROS. Confocal microscopy images revealed that MitoSOX fluorescence was highly co-localized with mitochondrial structures after irradiation, confirming the mitochondrial origin of the ROS. Quantitative analysis revealed a significant dose-dependent increase in mtROS. Compared with those of the control, the mean fluorescence intensity (MFI) values increased by 1.8-fold at 5 Gy, 3.2-fold at 10 Gy, and 5.6-fold at 15 Gy (Fig. 4c, d). These data confirm that radiation not only generates cytoplasmic ROS via water radiolysis but also directly triggers a mtROS burst. This mitochondrial damage event subsequently activates the opening of mitochondrial permeability transition pores (mPTPs), leading to the release of mtDNA into the cytoplasm.

Concurrently, DNA damage was observed, with the number of γ-phosphorylated histone H2AX (γ-H2AX) foci increasing 5.1-fold (Supplementary Fig. 2b, c). Radiation exposure initiated a self-perpetuating inflammatory cascade characterized by the sustained overexpression of iNOS (Fig. 4e, f) and the concentration of NO in the supernatant (Fig. 4g). The levels of inflammatory cytokines secreted by irradiated cells subsequently increase. This manifested as an 8.2-fold increase in tumor necrosis factor-alpha (TNF-α) (Fig. 4h), a 5.4-fold increase in interleukin-6 (IL-6) (Fig. 4i), and a 6.3-fold increase in interferon-gamma (IFN-γ) (Fig. 4j). The conditioned media from irradiated microglia-induced significant neuronal loss in cultures of murine hippocampal neuronal cell line (HT22) (Supplementary Fig. 2a). The number of NeuN-positive cells decreased by 58.8% (*p* < 0.001; Fig. 4k, l). These findings indicate that microglial activation initiates the hippocampal degeneration observed in vivo. Notably, the correlation between TNF-α protein and mRNA levels, along with the dose‒response consistency, validated the cascading pathology (Supplementary Fig. 2d, e).

In conclusion, excessive ROS generation is a key driver of inflammation-induced neurodegenerative pathologies. Our integrated analysis systematically demonstrated a dose-dependent hormetic effect of ionizing radiation. Specifically, low-dose irradiation (5 Gy) elicits protective hormetic stress. This moderate oxidative challenge may activate the Nrf2/Keap1-HO-1 signaling axis and upregulate vitagenes, thereby enhancing cellular antioxidant defenses and DNA repair mechanisms.^41^ This adaptive response confers a “preconditioning effect” that mitigates oxidative damage. Conversely, high-dose irradiation (15 Gy) exceeds the hormetic protective threshold. The resulting overwhelming ROS accumulation surpasses the compensatory capacity of the Nrf2 network, leading to irreversible oxidative injury, robust neuroinflammation, and widespread apoptosis, which are pathophysiological hallmarks of RIBI.^42^

### Radiation-induced mitochondrial DNA leakage activates microglial cGAS-STING neuroinflammation

We further investigated the detailed mechanisms underlying microglia-mediated neuroinflammatory cascades. Radiation-induced mitochondrial dysfunction has emerged as the critical pathophysiological nexus bridging acute oxidative stress to chronic neuroinflammation.^43^ Mechanistically, ionizing radiation induces structural and functional alterations in mitochondria through energy deposition. Excessive ROS generated by the dysfunctional electron transport chain act as genotoxic agents, leading to double-strand breaks in mtDNA.^44^ Disruption of the mitochondrial crista structure and alterations in the membrane potential facilitate the leakage and subsequent release of damaged mtDNA.^45,46^ These ROS bursts not only cause lipid peroxidation and protein carbonylation but also promote the opening of the mPTP, facilitating the cytosolic leakage of mtDNA fragments and cytochrome C.^47–49^

**Fig. 5:**
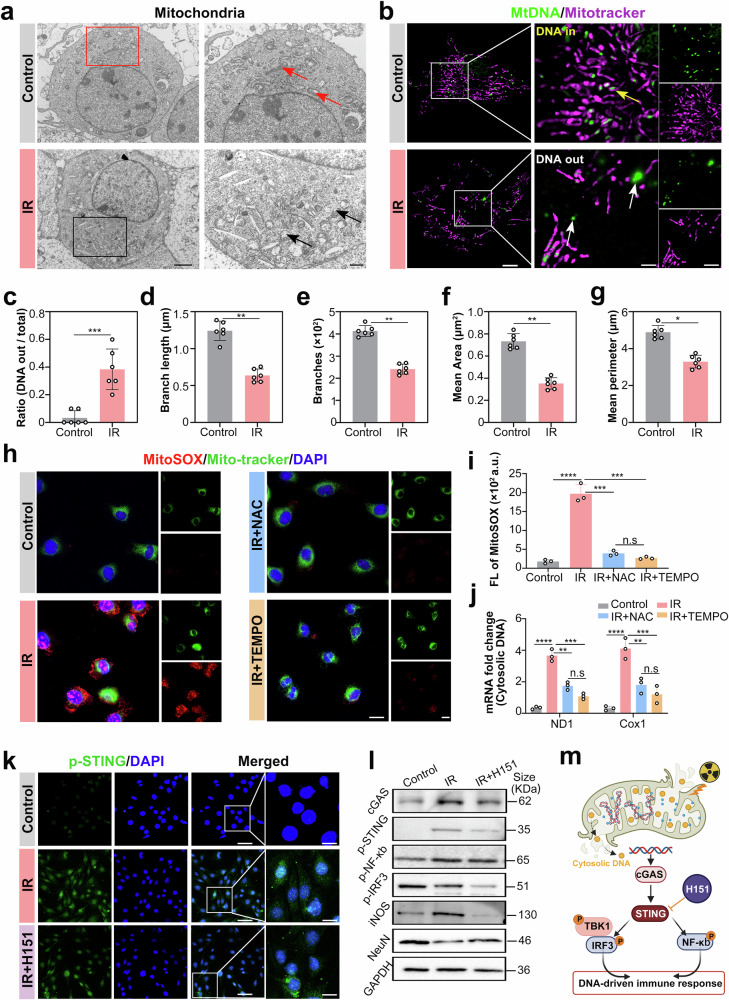
Radiation-induced mitochondrial DNA leakage activates microglial cGAS-STING neuroinflammation. **a** Representative TEM images of mitochondria in control and 15 Gy-irradiated BV2 cells. Higher-magnification images revealed crista fragmentation (black arrowheads) and matrix vacuolization in irradiated mitochondria, in contrast to the intact crista architecture in the control (red arrowheads). **b** Live-cell mitochondrial DNA visualization (MitoTracker: PK Mito Deep Red, MtDNA: SYBR; yellow arrows: cristae-localized mtDNA; white arrows: cytosolic mtDNA). **c** Quantification of mtDNA leakage frequency (*n* = 6 cells/group; Student’s *t* test). **d**–**g** Mitochondrial morphology analysis: Branch length (**d**), number of branches (**e**), mean area (**f**), and mean perimeter (**g**) (*n* = 6 mitochondria; Student’s *t* test). **h, i** Representative confocal images and quantification of relative fluorescence intensity of BV2 cells co-stained with MitoSOX Red (mtROS, red) and MitoTracker Green (mitochondria, green) at 6 h post-irradiation. BV2 cells were pretreated with NAC (10 mM) or MitoTEMPO (5 μM) for 1 h. **j** Cytosolic mRNA levels of the mtDNA-encoded genes ND1 and Cox1 (normalized to 18S rRNA). **k** p-STING immunofluorescence images of the control, irradiated (15 Gy), and irradiated + H151 (STING inhibitor) groups (nuclei: DAPI; STING: Alexa Fluor 488). **l** Western blot analysis of cGAS-STING pathway proteins (cGAS, p-STING, p-NF-κB, p-IRF3), inflammatory and neuronal markers (iNOS, NeuN), and GAPDH as a loading control. **m** Schematic diagram illustrating the mechanism by which radiation-induced mtDNA leakage triggers cGAS-STING-mediated neuroinflammatory cascades. Image was created in BioRender. Shang, Y. (2025) https://BioRender.com/mo dthph (agreement number: LW28JKG73J). All the statistical data are presented as the means ± SEMs. Scale bars: 2 μm (**a**), 5 μm (**b**, Whole mitochondria), 1 μm (**b**, insets), and 20 μm (**h**, **k**)

Transmission electron microscopy (TEM) analysis revealed that compared with control microglia, irradiated microglia presented pronounced ultrastructural mitochondrial abnormalities (indicated by red arrows), characterized by swollen mitochondrial morphology and crista disruption (denoted by black arrows) (Fig. 5a). Unirradiated mitochondria exhibited a characteristic elongated morphology with an intact double-membrane architecture, densely packed cristae, and a homogeneous electron-dense matrix. Postirradiation pathological remodeling features disrupted membrane continuity with localized protrusions and vacuole formation; fragmented or completely collapsed cristae displaying disordered stacking patterns; and markedly reduced matrix electron density.^50^ Superresolution microscopy revealed intramitochondrial crista collapse and fragmentation within irradiated microglia (Supplementary Movie 1), indicating that mitochondrial structural failure is a potential initiating event in neuroinflammatory cascades.

Under physiological conditions, mitochondria in microglia maintain a dynamic network with intact double-membrane structures and tightly packed cristae, and mtDNA is predominantly localized within the matrix or cristae junctions (Fig. 5b, yellow arrows). This architecture supports efficient oxidative phosphorylation and prevents mtDNA from being exposed to the cytosol. Radiation-induced overproduction of ROS disrupted mitochondrial homeostasis, leading to cristae remodeling and mtDNA leakage into the cytosol (Fig. 5b, white arrows; Supplementary Movie 2). Compared with those under the control conditions, the proportion of cells exhibiting cytosolic mtDNA leakage significantly increased to 43.7% (*p* < 0.001; Fig. 5c). Radiation exposure induced profound mitochondrial fragmentation in BV2 microglia, as evidenced by quantitative analysis of key morphometric parameters. Compared with control cells (branch length: 1.3 ± 0.2 μm; junctions: 402 ± 12/cell; area: 0.75 ± 0.09 μm²; perimeter: 5.3 ± 0.5 μm), irradiated cells (15 Gy) presented a 43% reduction in branch length (*p* < 0.001, Fig. 5d), a 32% loss of network junctions (*p* < 0.01, Fig. 5e), a 36% decrease in the mean mitochondrial area (p < 0.01, Fig. 5f), and a 25% shortening of the perimeter (*p* < 0.05, Fig. 5g). These coordinated structural alterations, marked by shortened tubules, simplified connectivity, and compacted organelles, reflect a radiation dose-dependent shift from interconnected networks to fragmented puncta, which is consistent with the crista disassembly observed via TEM.

To further substantiate the causal relationship between ROS and mtDNA release, BV2 cells were pretreated with the ROS scavenger NAC (10 mM) and the mitochondrial ROS scavenger MitoTEMPO (5 μM). MitoSOX detection confirmed that mitochondrial ROS levels were suppressed by 62% and 69% after irradiation (Fig. 5h, i). Nuclear contamination-free cytosolic isolation technology (FastPure Cell DNA isolation kit, nuclear gene GAPDH Ct > 32) combined with mtDNA-ND1 and Cox1 qPCR analysis was used. In the irradiation group, the expression of ND1 in the cytosolic mtDNA increased 3.8-fold compared with that in the control group, whereas that in the NAC and MitoTEMPO groups increased only 1.7-fold and 1.1-fold, respectively. The expression of Cox1 in cytosolic mtDNA increased to 4.2-fold that of the control, whereas that in the NAC and MitoTEMPO groups increased to only 2.2-fold and 1.3-fold, respectively (Fig. 5j), confirming that ROS scavenging blocks cytosolic mtDNA translocation. These findings directly demonstrate that a ROS burst is necessary for mtDNA leakage, further reinforcing the causal relationship between ROS and mtDNA release.

Crucially, this morphological collapse directly facilitated mtDNA leakage by destabilizing cristae junctions that normally sequester mitochondrial genomes. Leaked mtDNA activated the cGAS-STING pathway, as evidenced by STING initiation (Fig. 5k and Supplementary Fig. 3a) and downstream phosphorylation (p-NF-κB and p-IRF3). Strikingly, the inhibition of STING with H151 significantly attenuated the expression of neuroinflammatory markers (iNOS was reduced by 52%, *p* < 0.01) and mitigated neuronal loss (NeuN recovery increased by 28%, *p* < 0.05), thereby mechanistically linking mitochondrial genome instability to cytokine-driven neurodegeneration (Fig. 5l and Supplementary Fig. 3b).

Triggered by mtDNA leakage, the NLRP3 inflammasome, TLR9 and cGAS-STING innate immune pathways are concomitantly activated.^51^ We implemented a pharmacological coinhibitory strategy targeting all three pathways via MCC950 (NLRP3i, 10 μM), ODN 2088 (TLR9i, 5 μM), and H151 (STINGi, 1 μM). Key findings revealed that single-pathway inhibition reduced proinflammatory cytokines (IL-6/TNF-α/IL-1β) by 35% on average, whereas triple inhibition synergistically enhanced the anti-inflammatory effect, with cytokine levels decreasing by 56%. Strikingly, STING inhibition exclusively suppressed IFN-γ, whereas NLRP3i/TLR9i failed to significantly alter IFN-γ levels (Supplementary Fig. 3c). These results demonstrate that the NLRP3/TLR9 and cGAS-STING pathways synergistically drive cytokine storms that disrupt neural homeostasis. The STING–IFN-γ axis has emerged as a critical amplifier driving the relentless progression of neuroinflammation.

In summary, the schematic model depicted in Fig. 5m illustrates how radiation-induced mtDNA leakage activates the cGAS-STING neuroinflammatory cascade. Radiation exposure triggers damage to mitochondrial cristae in microglia, leading to structural collapse of the inner mitochondrial membrane. This disruption facilitates the release of mtDNA from cristae junctions into the cytosol, as indicated by black arrows. Cytosolic mtDNA binds to cGAS, initiating cGAMP synthesis and subsequent activation of the STING protein. Activated STING recruits and phosphorylates downstream effectors, including NF-κB and IRF3, driving the transcription of proinflammatory mediators. The use of the STING inhibitor H151 effectively blocks this signaling cascade, confirming the critical role of the cGAS-STING axis in linking mitochondrial damage to neuroinflammation.

### Characterization and in vivo validation of the Pep-Cu_5.4_O@H151 cascade-targeting nanotherapeutic

**Fig. 6:**
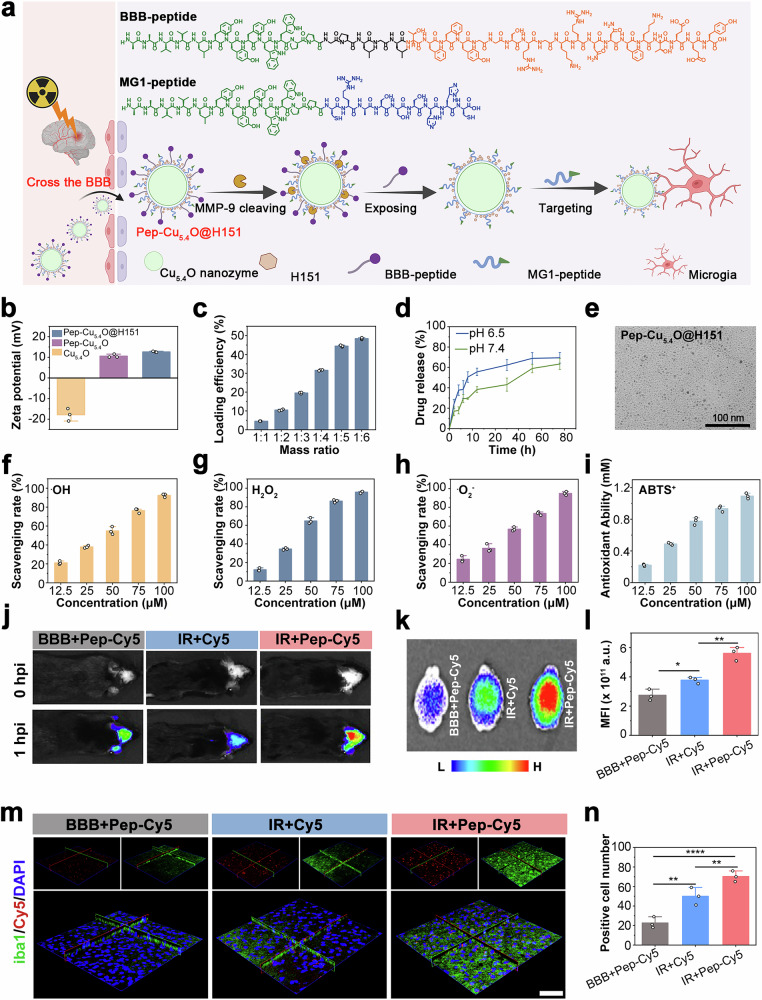
Characterization and in vivo validation of the Pep-Cu_5.4_O@H151 cascade-targeting nanotherapeutic. **a** Schematic of cascade targeting and peptide sequences: Ionizing radiation disrupts BBB integrity, enabling BBB penetration and subsequent MMP-9-responsive activation at neuroinflammatory lesions. MMP-9 cleaves the shielding peptide to expose targeting motifs for activated M1 microglia. The BBB peptide sequence is PPWWYYYLVVAA-LGLPG-YEETKFNNRKGRSGGYFF, and the MG1 peptide sequence is PPWWYYYLVVAA-CHHSSSARC. The peptide sequences were designed in ChemDraw software, and the scheme image was created in BioRender. Shang, Y. (2025) https://BioRender.com/h2 5oed1 (agreement number: DV28JKG5ZS). **b** Z-potential measurements confirming surface charge modification. **c** Drug-loading efficiency at various precursor ratios. **d** pH-dependent drug release profiles (pH = 6.5, 7.4). **e** TEM image of Pep-Cu_5.4_O@H151. **f**–**i** ROS scavenging capacity: Hydroxyl radical (•OH, **f**), hydrogen peroxide (H_2_O_2_, **g**), superoxide anion (O₂^−^, **h**), and total ROS elimination (**i**). **j** Whole-body fluorescence imaging showing time-dependent brain accumulation after intravenous injection in mice. **k, l** Ex vivo brain imaging (**k**) and quantitative ROI analysis (**l**, *n* = 3 mice; one-way ANOVA). **m**, **n** Immunofluorescence image of a brain section showing the colocalization of Cy5 (red) with iba1^+^ microglia (green) (**m**), and the results were quantified (**n**, one-way ANOVA). All the statistical data are presented as the means ± SEMs. Scale bar: 100 nm (**e**); 20 μm (**m**)

Given the aforementioned findings, we successfully developed a cascade-targeting peptide nanodrug (Pep-Cu_5.4_O@H151) with the ability to mitigate neuroinflammation in the context of radiation-induced injury, which was specifically designed to precisely target M1-polarized microglia activated by radiation-induced brain damage. The design of this nanodrug is based on the principle that mitochondrial ROS play a central role in triggering mtDNA leakage and that STING signaling is causally involved in perpetuating neuroinflammation. Specifically, Pep-Cu_5.4_O@H151 was synthesized via the use of self-assembling peptides as templates to construct nanozymes, followed by subsequent loading of the cGAS-STING axis inhibitor H151 into the BBB, which allows the nanodrug to capitalize on the specificity of the inflammatory microenvironment. Upon crossing the BBB and reaching inflammatory foci, locally overexpressed matrix metalloproteinase-9 (MMP-9) selectively cleaves long-chain BBB-targeting peptides. Thus, the BBB-targeting peptide was designed to incorporate multiple critical structural motifs, including a low-density lipoprotein receptor (LDLR)-binding sequence (YEETKFNNRKGRSGGYFF), an MMP-9-cleavable sequence specifically recognized by inflammation-overexpressing MMP-9 (LGLPG), and a hydrophobic metal-binding peptide (PPWWYYYLVVAA). Concurrently, the MG1 peptide consists of a sequence targeting M1 microglia activated after brain injury and a hydrophobic sequence for metal ion coordination (PPWWYYYLVVAA-CHHSSSARC).^52,53^ This sequence exposes cryptic short-chain targeting moieties. These activated epitopes precisely recognize activated M1-polarized microglia, enabling spatiotemporally controlled payload delivery. Crucially, following successful internalization into M1-activated microglia, the Cu_5.4_O nanozyme rapidly initiates intracellular radiation-triggered ROS scavenging while simultaneously orchestrating sustained release of the cGAS-STING pathway inhibitor H151. This dual-modality mechanism effectively attenuates pathological microglial hyperactivation, ultimately achieving targeted therapeutic effects against radiation-induced brain injury (Fig. 6a). We successfully synthesized ultrasmall Cu_5.4_O nanozymes via a one-step L-ascorbic acid reduction method. TEM images revealed monodisperse spherical nanoparticles with an average diameter of 2.5 ± 0.3 nm (Supplementary Fig. 4a). X-ray photoelectron spectroscopy (XPS) quantitative deconvolution of the Cu 2*p* peak areas revealed a normalized mass ratio of 3:4 for the Cu to Cu₂O phases (Supplementary Fig. 4b, c). On the basis of this stoichiometric analysis, the nanoparticles were designated as Cu_5.4_O nanozymes. Moreover, we designed two functional peptides to further enhance the ability of the nanodrugs to cross the BBB and target microglia (Supplementary Fig. 4d, e). Furthermore, LC‒MS analyses also confirmed that MMP-9-mediated specific proteolytic cleavage of functional peptide 1 (Supplementary Fig. 4f). Coassembly of these two peptides with Cu_5.4_O nanozymes at pH 7 yielded Pep-Cu_5.4_O nanozymes. During the experiment, as the doping ratio of the peptide increased, flocculent precipitates gradually appeared in the solution. This phenomenon is most likely due to the chelation between copper and the polypeptide. (Supplementary Fig. 4g, h). TEM characterization revealed that the Pep-Cu_5.4_O nanozymes had a diameter of <5 nm and were monodisperse (Supplementary Fig. 4i). The success of comineralization was confirmed by the inversion of the zeta potential. Specifically, zeta potential measurements revealed a shift from a negative charge for Cu_5.4_O (−17.82 mV) to a positive charge for Pep-Cu_5.4_O (+10.60 mV). This charge transition was attributed primarily to the cationic charge of the targeting peptide (Fig. 6b and Supplementary Fig. 4j). FTIR spectroscopy revealed characteristic amide I/II band shifts (Supplementary Fig. 4k). We subsequently coassembled the cGAS–STING pathway inhibitor H151 with Pep-Cu_5.4_O nanozymes via hydrophobic interactions to form Pep-Cu_5.4_O@H151 nanodrugs. A systematic investigation of drug-loading parameters revealed a dose-dependent increase in H151 loading efficiency, reaching a plateau at 44% with a 1:5 mass ratio of H151 to Pep-Cu_5.4_O nanozymes (Fig. 6c). Drug release profiling under physiological (pH = 7.4) and inflammatory (pH = 6.5) conditions demonstrated sustained H151 release kinetics (Fig. 6d). The experimental results showed that Pep-Cu_5.4_O@H151 could release the H151 inhibitor slowly under both pH conditions, a characteristic that enables its effective application across diverse in vivo microenvironments. Additionally, nanoparticle size and targeting performance represent critical determinants of in vivo therapeutic efficacy. TEM imaging confirmed that Pep-Cu_5.4_O@H151 maintained a sub-5 nm particle size (Fig. 6e), a nanoscale dimension essential for biological diffusion and targeted transport. These findings collectively validated the successful synthesis of our designed Pep-Cu_5.4_O@H151 nanodrug.

To systematically evaluate the ROS-scavenging capacity of Pep-Cu_5.4_O@H151 nanodrugs, we conducted comprehensive analyses targeting four representative reactive oxygen species: hydroxyl radicals (•OH), hydrogen peroxide (H₂O₂), superoxide anions (O₂^−^), and total ROS. The nanodrugs exhibited concentration-dependent scavenging efficacy across all the tested species. Notably, at 100 μg/mL, Pep-Cu_5.4_O@H151 demonstrated robust ·OH neutralization exceeding 90% (Fig. 6f and Supplementary Fig. 4l) while achieving decomposition of ~96% H₂O₂ at the same concentration (Fig. 6g and Supplementary Fig. 4m). Strikingly, even at 50 μg/mL, the formulation effectively scavenged 50% of the O₂⁻ species (Fig. 6h and Supplementary Fig. 4n). Further validation through an ABTS⁺ radical cation assay revealed exceptional antioxidant capacity, with 75 μg/mL Pep-Cu_5.4_O@H151 exhibiting Trolox-equivalent antioxidant activity (95.64 mM Trolox control reference, Fig. 6i). Quantitative comparisons demonstrated superior ROS scavenging performance across all four detection systems. These findings collectively establish Pep-Cu_5.4_O@H151 as a potent radiation countermeasure capable of executing efficient ROS neutralization cascades, preserving mitochondrial DNA integrity via oxidative damage mitigation, and suppressing neuroinflammation through redox homeostasis restoration.

To further validate the brain-targeting efficacy of Pep-Cu_5.4_O@H151 nanodrugs, we utilized a localized RIBI murine model. At the mid-treatment phase (day 7 postirradiation), intravenous administration of free Cy5 and Cy5-labeled peptides was performed. Whole-body fluorescence imaging revealed rapid cerebral accumulation within 1 hpi, with signal clearance observed by 8 hpi (Fig. 6j and Supplementary Fig. 5a). The results of the quantitative analysis revealed a 1.6-fold increase in cerebral signal intensity in the mice that received Pep-Cy5 compared with the free Cy5 controls (*p* < 0.05). Notably, the red fluorescence intensity in the RIBI model group was 2.1-fold greater than that in the healthy control group (Fig. 6k, l), further demonstrating that ionizing radiation-induced neuroinflammatory microenvironments enhance the targeting specificity of nanodrugs. Histological evaluation of brain sections revealed preferential nanodrug accumulation in iba1^+^-activated microglial regions, as confirmed by Cy5 colocalization analysis (Fig. 6m). This microglial-targeting specificity was 1.5-fold greater than that of free Cy5 (*p* < 0.01, Fig. 6n). To validate the brain-targeting efficiency of the material, inductively coupled plasma‒mass spectrometry (ICP-MS) was used to quantitatively detect copper accumulation in major organs. The results revealed that brain copper accumulation in the targeted peptide-modified group (IR + Pep-Cu_5.4_O@H151) reached 19.5 ± 0.3 ID/g, which was significantly greater than that in the nontargeted group (IR + Cu_5.4_O, 14.7 ± 1.2 ID/g). Moreover, substantial copper accumulation was observed in kidney tissues, which was significantly greater than that in the liver and spleen and was directly associated with the hydrated particle size (<5.5 nm) of the material. This size range allows nanoparticles to be excreted through glomerular filtration into the urine. No significant difference in copper accumulation was found between the two groups in other organs, such as the heart and lung, indicating that targeted modification did not cause systemic distribution deviation (Supplementary Fig. 5b). In summary, we successfully engineered an excellent BBB-penetrating and microglia-targeted nanodrug for precise intervention in radiation-induced neuroinflammation.

The safety profile and biocompatibility of nanomaterials are critical prerequisites for their biomedical applications. In vitro cytotoxicity assessment via CCK-8 assays revealed negligible toxicity of Pep-Cu_5.4_O@H151. The cell viability remained above 70%, even at the highest tested concentration (400 μg/mL) (Supplementary Fig. 6). To further validate biosafety in vivo, we employed a zebrafish embryo model. Embryos at 3 hpf were exposed to increasing doses of Pep-Cu_5.4_O@H151 (20, 100, 400 μg/mL). Phenotypic monitoring at critical developmental stages (24, 48, 72 hpf) revealed no morphological abnormalities or developmental delays across all treatment groups (Supplementary Fig. 7a). Quantitative analysis confirmed comparable survival rates at 24 hpf (*p* > 0.05, Supplementary Fig. 7b) and hatching rates at 72 hpf (*p* > 0.05, Supplementary Fig. 7c), demonstrating excellent embryonic biocompatibility.

Systemic safety was further verified in murine models. Hemolysis assays revealed no significant erythrocyte lysis (<2% hemolytic ratio) at concentrations up to 1000 μg/mL (Supplementary Fig. 8a, b). Comprehensive hematological profiling revealed normal ranges for key parameters: white blood cell (WBC) count, hemoglobin concentration (HGB), hematocrit (HCT), mean corpuscular hemoglobin (MCH), and mean corpuscular volume (MCV) (Supplementary Fig. 8c–g). Histopathological examination of major organs (heart, liver, spleen, lungs, and kidneys) via HE staining confirmed the absence of inflammatory infiltration, necrosis or architectural disruption (Supplementary Fig. 8h).

### In vitro therapeutic effects of Pep-Cu_5.4_O@H151 nanodrugs on RIBI

**Fig. 7:**
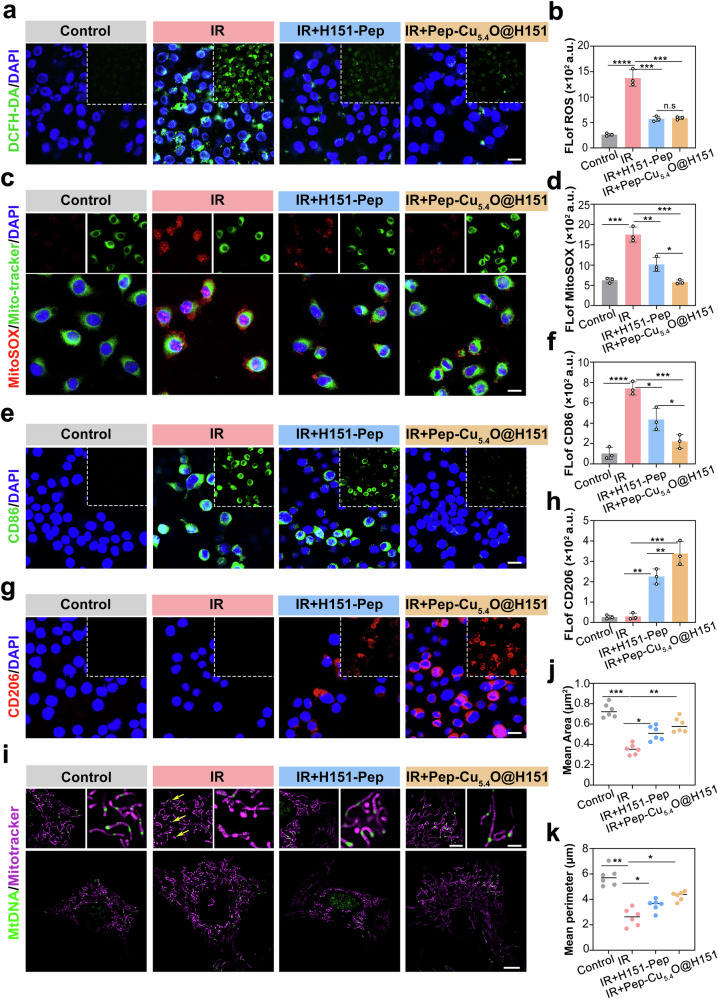
In vitro therapeutic effects of Pep-Cu_5.4_O@H151 nanodrugs on RIBI. **a** Representative images of intracellular ROS detection via the DCFH-DA fluorescent probe in BV2 cells. **b** Quantification of ROS fluorescence intensity (*n* = 3 fields; one-way ANOVA). **c** Representative images of mtROS detection via the MitoSOX fluorescent probe in BV2 cells. **d** Quantification of mtROS fluorescence intensity (*n* = 3 fields; one-way ANOVA). **e** Immunofluorescence images of M1-polarized macrophages stained for CD86 (Alexa Fluor 488, green). **f** Quantitative analysis of CD86 fluorescence intensity normalized to that of DAPI (*n* = 5 fields; one-way ANOVA). **g** Immunofluorescence images of M2-polarized macrophages stained for CD206 (Alexa Fluor 594, red). **h** Quantitative analysis of CD206 fluorescence intensity normalized to that of DAPI (*n* = 5 fields; one-way ANOVA). **i** SYBR Gold-enhanced superresolution imaging of live HT22 mitochondria in culture medium. **j**, **k** Mitochondrial morphology analysis of HT22 cells: mean area (**j**) and mean perimeter (**k**) (*n* = 6 mitochondria; Student’s *t* test). All the statistical data are presented as the means ± SEMs. Scale bars: 20 μm (**a**, **c**, **e**, and **g**), 10 μm (**h**)

Leveraging the dual functionality of the nanomaterial, we initiated therapeutic validation using an in vitro microglia–neuron coculture system to assess neuroprotective efficacy. As anticipated, H151-Pep effectively mitigated radiation-induced ROS bursts in BV2 microglia (Fig. 7a), reducing intracellular ROS levels by 42.3% compared with those in the irradiation group. The Pep-Cu_5.4_O@H151 nanodrug formulation also exhibited suppression (43.5% reduction in ROS). However, no statistically significant difference was detected between the H151-Pep and Pep-Cu_5.4_O@H151 groups (*p* > 0.05, Fig. 7b). Given this, we further employed a MitoSOX fluorescent probe to detect mtROS levels, followed by correlation analysis with cytoplasmic ROS data from DCFH-DA assays (Fig. 7c). As shown in Fig. 7c, d, mtROS levels in the nanodrug treatment group were 45.2% lower than those in the radiation control group, significantly outperforming those in the H151-Pep monotherapy group (32.1%, *p* < 0.05; Fig. 7d). This finding is closely associated with the mitochondrial protective function of nanodrugs, which suppress mtROS bursts by targeting the mitochondrial source.

Similarly, radiation exposure led to a significant increase in the number of proinflammatory M1 macrophages. However, the introduction of H151 induced macrophage polarization from the proinflammatory M1 phenotype to the reparative M2 phenotype. Compared with that in the H151 group, the CD86⁺ signal intensity in the Pep-Cu_5.4_O@H151 nanodrug group decreased 2.1-fold, whereas the CD206⁺ signal intensity increased 3.6-fold (Fig. 7e–h). These findings indicate that the combination of H151 and Cu_5.4_O nanozymes had optimal effects on promoting macrophage polarization from the M1 phenotype to the M2 phenotype. This polarization shift from M1 to M2 macrophages by Cu_5.4_O@H151 nanodrugs effectively protected mitochondria from radiation-induced damage in HT22 neurons, reversing radiation-induced mitochondrial fragmentation (Fig. 7i, yellow arrows). This was evidenced by increases in the mean area (Fig. 7j) and mean perimeter (Fig. 7k).

In summary, our cellular studies demonstrated that Pep-Cu_5.4_O@H151 nanodrugs significantly attenuated radiation-induced neuronal damage through coordinated modulation of microglial polarization and mitochondrial protection. Notably, while H151-Pep moderately reduces secondary ROS via inflammatory pathway inhibition, Pep-Cu_5.4_O@H151 achieves primary ROS elimination coupled with upstream STING suppression. This dual-action mechanism explains its greater mitochondrial protection, which is critical considering that ROS-induced mtDNA leakage perpetuates cGAS–STING activation.

### Therapeutic efficacy and long-term neuroprotection of the Pep-Cu_5.4_O@H151 nanodrug

**Fig. 8:**
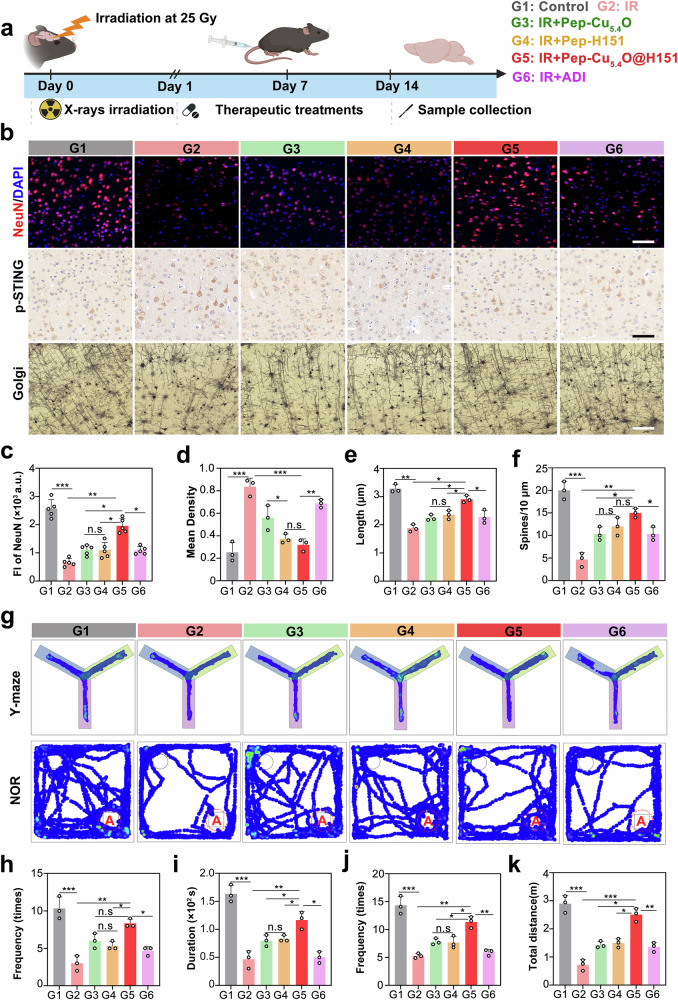
In vivo therapeutic effects of Pep-Cu_5.4_O@H151 nanodrugs on RIBI. **a** Schematic illustration of the in vivo experimental design. The mice were exposed to fractionated radiation (5 Gy × 5 times, cumulative 25 Gy) to induce RIBI. Starting 24 h post-IR, the mice were administered 150 μL of the test formulations via tail vein injection daily for 14 consecutive days. The experimental groups included the following: (1) control (PBS injection, G1); (2) IR group (radiation, G2); (3) Pep-Cu_5.4_O group (radiation + 5 mg/kg Pep-Cu_5.4_O, G3); (4) Pep-H151 group (radiation + 5 mg/kg Pep-H151, G4); (5) Pep-Cu_5.4_O@H151 group (radiation + 5 mg/kg total concentration, with 2.5 mg/kg Pep, 2.5 mg/kg Cu_5.4_O, and 1.25 mg/kg H151, G5); and (6) ADI group (radiation + 10 mg/kg idebenone as a positive control, G6). Each group consisted of six mice. **b** Representative NeuN immunofluorescence staining images (Alexa Fluor 594, red) and representative STING immunohistochemistry (DAB) images of the cortex. Images of Golgi-Cox-stained pyramidal neurons. **c** Quantification of NeuN⁺ fluorescence intensity normalized to that of DAPI⁺-stained nuclei (*n* = 5 fields; one-way ANOVA). **d** Quantification of the mean STING⁺ density (*n* = 3 fields; one-way ANOVA). **e**, **f** Dendritic complexity analysis via the Sholl assay: radial length (**e**) and branch density (**f**) (*n* = 3 fields; one-way ANOVA). **g** Representative heatmaps of Y-maze and novel object recognition tests in 16 wpi mice. **h** Frequency of entries into the novel arm (N) in the Y-maze test. **i** Duration of time spent in the novel arm (N) of the Y-maze. **j** Frequency of interactions with the novel object (Object A) during the novel object recognition test. **k** Total movement distance across the testing area. All the statistical data are presented as the means ± SEMs. Scale bar: 20 μm (**b**)

In view of the remarkable in vitro therapeutic efficacy of the Pep-Cu_5.4_O@H151 nanodrug in treating RIBI, we conducted in vivo efficacy evaluations using a clinically relevant murine RIBI model. The experimental design was as follows: a fractionated irradiation protocol of 5 Gy × 5 times (cumulative 25 Gy, biological effective dose (BED) = 43.75 Gy, *α*/*β* = 2 Gy) was employed, with daily tail–vein injections administered for 14 consecutive days. This dosage regimen mimics the fractionated irradiation pattern used in clinical adjuvant postoperative radiotherapy, and its BED value is equivalent to that of moderate-dose protocols for head and neck tumor radiotherapy. We established six experimental groups (*n* = 6/group): control (PBS), irradiated (IR, 25 Gy), IR + Pep-H151 (5 mg/kg), IR + Pep-Cu_5.4_O (5 mg/kg), IR + Pep-Cu_5.4_O@H151 (5 mg/kg), and IR + idebenone (ADI, 10 mg/kg) as a clinically validated positive control drug (Fig. 8a).

To comprehensively evaluate the antineuroinflammatory efficacy of our engineered nanoformulation, we implemented a multiscale assessment protocol targeting acute pathological resolution and long-term functional recovery. At the 14-day treatment endpoint, Pep-Cu_5.4_O@H151 demonstrated superior neuronal restoration, achieving 89.7% cortical NeuN⁺ neuron density recovery compared with that of healthy controls. These values were 1.4-fold and 1.5-fold greater than those of the single-agent Pep-Cu_5.4_O and Pep-H151 therapies, respectively, while exceeding the clinical antioxidant ADI by >25% (Fig. 8b, c). p-STING/immunoreactivity detection has revealed that H151 is an anti-inflammatory target. In the irradiated group, STING phosphorylation was 3.2-fold greater than that in the control group. However, Pep-Cu_5.4_O@H151 decreased by 78%, which mechanistically confirmed H151-mediated blockade of the cGAS-STING-driven neuroinflammatory cascade (Fig. 8b, d). Most importantly, the expression analysis of cGAS-STING pathway proteins in mouse brain tissues provided robust mechanistic evidence for the therapeutic efficacy of Pep-Cu_5.4_O@H151. Quantitative Western blotting revealed that irradiation induced a 2.8-fold increase in cGAS protein expression, which was significantly suppressed by 25% in the Pep-Cu_5.4_O@H151 group. Concurrently, STING phosphorylation (p-STING Ser366) in the IR group increased 3.2-fold, which was reduced by 78% in the nanodrug group, confirming the inhibition of the cGAS-STING axis. Downstream signaling analyses revealed that irradiation triggered a 2.6-fold increase in p-IRF3 (Ser386) and a 1.6-fold increase in p-NF-κB p65 (Ser536) levels, whereas Pep-Cu_5.4_O@H151 treatment attenuated these increases by 32% and 26%, respectively. Neuronal integrity assessments revealed that the NeuN density in the Pep-Cu_5.4_O@H151 group was restored to 89.7% of that in the control group, with a significant positive correlation between NeuN expression and p-STING reduction (Supplementary Fig. 9a). These integrated results demonstrate that Pep-Cu_5.4_O@H151 exerts neuroprotective effects via dual inhibition of cGAS-STING pathway activation and downstream inflammatory cascades, thereby promoting neuronal survival and functional recovery.

Golgi staining revealed markedly greater dendritic complexity in the nanodrug group than in the irradiated group, with a 1.8-fold increase in the number of dendritic intersections within a 10 μm radius and a 2.1-fold increase in the mushroom-shaped spine density, suggesting enhanced synaptic remodeling (Fig. 8b, e, f). HE staining of the cortex revealed restored neuronal alignment. In the hippocampal DG region, the density of Nissl bodies was 42 ± 1.2 per region, which was 1.4-fold greater than that in the irradiated group (Supplementary Fig. 9b, c). These data confirm the synergistic mechanism of Pep-Cu_5.4_O@H151 : the ROS scavenging ability of the Cu_5.4_O nanoenzymes and the inhibition of the H151 STING pathway complement each other to repair the neuronal structure.

Critically, therapeutic benefits extended beyond the acute injury phase, as 16 wpi behavioral assays demonstrated sustained cognitive preservation. In the Y-maze test, compared with the IR group, the Pep-Cu_5.4_O@H151 nanodrug group presented a 1.8-fold increase in novel arm entries and a 42% prolongation of exploration time (Fig. 8g–i). Notably, in the novel object recognition task, the frequency of target arm entries increased by 35.2%, accompanied by a 56.3% increase in movement distance (Fig. 8g, j, k). These findings indicate that the nanodrug not only mitigates acute radiation-induced neuronal damage but also prevents long-term cognitive decline, presumably through its dual mechanisms of ROS scavenging and STING pathway inhibition.

In summary, our study demonstrated that the Pep-Cu_5.4_O@H151 nanodrug effectively alleviates radiation-induced brain injury through a dual-mechanism approach. By simultaneously inhibiting the cGAS-STING pathway to reduce neuroinflammation and ROS via Cu_5.4_O nanozymes, this nanodrug promotes neuronal survival, synaptic remodeling, and cognitive function recovery in mice. These effects are evidenced by significant improvements in neuronal density, dendritic complexity, and behavioral performance, outperforming both single-component treatments and the clinically used antioxidant idebenone.

### Neuroprotection by the Pep-Cu_5.4_O@H151 nanoplatform at the tumor-normal tissue interface during radiotherapy for glioblastoma

**Fig. 9:**
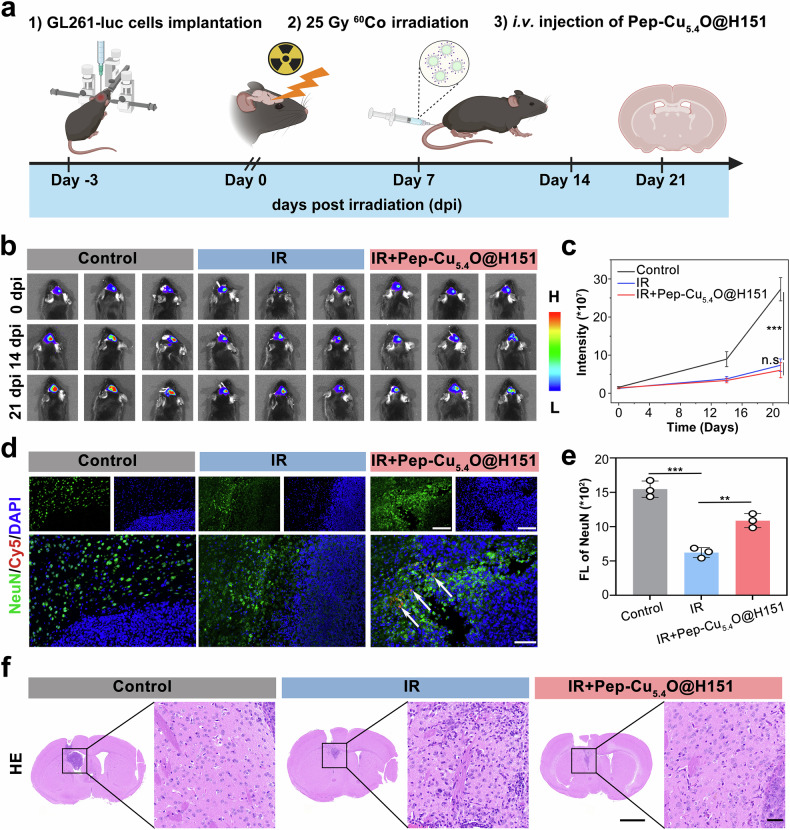
Neuroprotection by the Pep-Cu_5.4_O@H151 nanoplatform at the tumor–normal tissue interface during radiotherapy for glioblastoma. **a** Schematic illustration of the experimental design: The GL261 orthotopic glioma model was established via stereotactic inoculation, followed by fractionated radiotherapy (5 Gy × 5 days) and concurrent nanodrug administration (5 mg/kg, i.v., 14 days). **b** Representative in vivo bio-fluorescence images of tumor volume at 0, 14 and 21 days post-treatment. **c** Quantitative analysis of tumor bio-fluorescence intensity (*n* = 3 mice; one-way ANOVA). **d** Immunofluorescence staining of NeuN⁺ neurons. NeuN⁺ neurons (green); Pep-Cu_5.4_O@H151 nanodrugs (red, white arrows indicate neuron-specific accumulation); DAPI (blue). **e** Quantitative analysis of NeuN⁺ neuron fluorescence intensity (*n* = 3 fields; one-way ANOVA). **f** HE staining of mouse brains. The left panel shows the whole-brain image, and the right panel presents a magnified view of the area within the black box in the left panel. All the statistical data are presented as the means ± SEMs. Scale bars: 20 μm (**d**), 1000 μm (**f**, right), and 20 μm (**f**, left)

During radiotherapy for head and neck tumors (such as nasopharyngeal carcinoma and brain glioma), normal tissues adjacent to the tumor not only face radiation damage but also need to cope with microenvironmental interferences such as tumor cell-secreted factors and abnormal vascular networks.^54^ To simulate the adjuvant radiotherapy scenario for residual tumors after glioma surgery in the clinic (~60% of patients are at risk of residual tumor recurrence),^55^ we established a GL261 orthotopic glioma model simulating postoperative radiotherapy scenarios (Fig. 9a). Following stereotactic tumor inoculation, the mice received clinically relevant fractionated irradiation (5 Gy × 5 days) with concurrent intravenous Pep-Cu_5.4_O@H151 administration (5 mg/kg, 14 days). This strategy was designed to explore the therapeutic value of the nanodrug in the interactive microenvironment between tumor and normal tissues.

In vivo fluorescence imaging revealed no significant increase in tumor volume in either the radiotherapy alone group or the radiotherapy combined with nanodrug group within 14 days (*p* > 0.05), representing a 25% reduction compared with that in the untreated group (Fig. 9b, c). Extended observation at 21 days posttreatment further confirmed stable tumor volumes without significant enlargement in both groups. These findings definitively demonstrate that the nanodrug neither interferes with the antitumor efficacy of radiotherapy nor promotes tumor proliferation or recurrence, providing direct experimental evidence for the safety of combining neuroprotectants with clinical radiotherapy. Immunofluorescence analysis revealed that the density of NeuN⁺ neurons within the peritumoral region in the nanodrug group was significantly greater (61%) than that in the radiotherapy-alone group (Fig. 9d, e). Moreover, the red fluorescence signal of the nanomaterial was specifically distributed in peritumoral normal tissues (indicated by white arrows), demonstrating obvious colocalization with NeuN⁺ neurons. These results directly confirm that the nanodrug can accumulate precisely in neuronal regions, exerting protective effects by inhibiting radiation-induced neuroinflammatory cascades. HE staining results revealed that the radiotherapy alone group exhibited typical pathological features of radiation injury in the brain region adjacent to the tumor, including disorganized tissue architecture with widened extracellular space, neuronal nuclear pyknosis, infiltration of atypical tumor cells at the tumor margin, and disrupted microvascular structures. In contrast, radiotherapy combined with the nanodrug had significant protective effects on peritumoral normal tissues, with ordered neuronal arrangement, a reduced nuclear fragmentation ratio, clear tumor boundaries, and no invasive cell diffusion (Fig. 9f).

Overall, the Pep-Cu_5.4_O@H151 nanodrug not only ensures the antitumor efficacy of radiotherapy but also eliminates the risk of promoting tumor proliferation or recurrence while significantly ameliorating radiation-induced tissue damage. These findings provide direct experimental evidence for the clinical application of neuroprotectants combined with radiotherapy and hold promise for establishing a precision radiotherapy paradigm with dual effects of “tumor cell killing—peritumoral normal tissue protection”.

## Discussion

RIBI, characterized by chronic neuroinflammation-driven neurodegenerative processes, poses an increasingly prominent clinical challenge in oncological radiotherapy. Epidemiological data indicate that the cumulative incidence of RIBI following radiotherapy ranges from 1.9% to 24% in patients with nasopharyngeal carcinoma, glioma, and meningioma. However, current therapeutic strategies provide only transient symptomatic relief and fail to reverse permanent neurological damage. Furthermore, RIBI has distinct radiobiological features, namely, chronic radiation-triggered neuroinflammation accelerates the neurodegenerative cascade; nevertheless, its underlying mechanisms remain incompletely elucidated. Currently, the dynamic toxicological effects and underlying mechanisms of radiation-induced neuroinflammation across different developmental stages remain unclear, and neuroprotective strategies capable of precisely targeting neuroinflammation are lacking. Notably, the cGAS-STING pathway, a central regulatory hub of innate immunity, has been demonstrated to drive inflammatory cascades in neurodegenerative diseases such as Alzheimer’s disease and Parkinson’s disease. However, its mechanistic role in RIBI and its potential as a therapeutic target have not been systematically explored. This study aimed to elucidate how radiation triggers neuroinflammation via the mitochondrial DNA leakage-cGAS-STING axis and to develop targeted intervention strategies.

In this study, we established a multiscale experimental platform. This platform integrates zebrafish larvae (for developmental toxicity profiling), adult zebrafish (with a mature neurobehavioral repertoire), and murine models to systematically investigate radiation-induced toxicological cascades. The platform mechanistically links molecular lesions to functional behavioral deficits. This study reveals the detrimental impacts of ionizing radiation on motor coordination, phototactic responsiveness, exploratory drive, and cognitive learning capacity. Additionally, these findings suggest the potential role of ionizing radiation in the initiation of neurodegeneration. These cross-species validations collectively establish a standardized framework for evaluating RIBI. Furthermore, we elucidated the principal molecular pathway underlying radiation-triggered neuroinflammation. Radiation-generated ROS critically compromise mitochondrial integrity, inducing crista fragmentation and subsequent cytoplasmic leakage of mtDNA. This abnormal accumulation of dsDNA potently activates the cGAS-STING pathway. The mechanistic connection between oxidative stress and neuroimmune dysregulation establishes the “mitochondrial–immune axis” as a therapeutic paradigm for RIBI intervention. These findings provide a foundation for the development of targeted neuroprotective strategies. Driven by the biological mechanisms aimed at disrupting radiation-induced neuroinflammatory cascades, a targeted nanoplatform has emerged as a novel therapeutic approach because of the following advantages. First, modified nanoparticles undergo transcytosis to cross the BBB, thereby increasing drug accumulation in the brain.^56,57^ Nanocarriers enable the efficient delivery of otherwise incompatible drugs, overcoming challenges related to drug solubility and stability.^58^ Nanoparticles with high biocompatibility selectively accumulate in irradiated tissues through the enhanced permeability and retention (EPR) effect, minimizing off-target effects.^59^ The combined effect of these characteristics has made nanomedicine a primary approach for treating brain diseases. Finally, we designed a precision-targeted nanotherapy (Pep-Cu_5.4_O@H151) that enables dual-pathway intervention: quenching oxidative stress by catalytically scavenging ROS while silencing neuroinflammatory signaling through STING inhibition. An innovative intervention strategy of “inhibiting endogenous pathways and regulating the exogenous microenvironment” has been established.

This research delivers dual breakthroughs: mechanistically, it delineates the complete pathogenic sequence from radiation-induced mitochondrial ROS generation to cytosolic mtDNA leakage, aberrant cGAS-STING activation, and consequent neuroinflammation through multimodal validation, filling critical knowledge gaps in RIBI pathogenesis; therapeutically, our nanodrug pioneers a precision neuroprotection strategy through simultaneous implementation of causal ROS clearance and molecular STING inhibition, enabled by cell-specific peptide-targeting technology. Despite these encouraging results, several limitations warrant consideration. First, although nanodrugs have demonstrated favorable safety in preclinical studies, the potential long-term accumulation and associated toxicity of nanodrugs require rigorous evaluation prior to clinical translation. Second, our focus on cytokine-mediated neuroinflammation has not explored potential crosstalk with other STING-regulated processes, such as autophagy or pyroptosis, which may contribute to RIBI progression. Finally, clinical heterogeneity, including variations in radiotherapy protocols and patient-specific genetic factors (e.g., STING gene polymorphisms), could significantly impact therapeutic response and necessitate personalized dosing strategies.

These limitations, however, delineate compelling pathways for future research and clinical development. Therapeutically, the modular design of our nanoplatform permits adaptive modifications: replacing targeting peptides could redirect them to neurons or astrocytes for broader applications in stroke or traumatic brain injury. Combining H151 with mitochondrial antioxidants (e.g., MitoQ) may yield synergistic neuroprotection. Clinically, prophylactic administration prior to radiotherapy could preemptively disrupt neuroinflammatory cascades, while monitoring cerebrospinal fluid (CSF) mtDNA levels might serve as biomarkers for early RIBI detection and treatment response assessment. Mechanistic investigations should establish radiation dose thresholds for mtDNA leakage, examine the epigenetic regulation of sustained STING activation (e.g., histone lactylation), and validate findings in large animal models, such as nonhuman primates, to accelerate clinical readiness.

Overall, our study systematically elucidates the core mechanism of RIBI, which involves ionizing radiation-generated mitochondrial ROS-induced mtDNA leakage, thereby triggering excessive activation of the cGAS-STING pathway and initiating a neuroinflammatory cascade. Building upon this mechanism discovery, we innovatively developed a multifunctional nanodrug, Pep-Cu_5.4_O@H151, that concurrently scavenges ROS and inhibits the STING signaling pathway. This nanotherapeutic significantly mitigated neuroinflammation in a mouse RIBI model established with a simulated clinical radiation dose of 25 Gy. Our research achieves dual breakthroughs: first, we establish the pivotal regulatory role of the cGAS-STING pathway in RIBI, representing its inaugural mechanistic elucidation in this context; second, we successfully develop a dual-pathway interventional drug that targets both the etiology and molecular drivers of RIBI. This work not only provides a novel precision intervention strategy for RIBI but also pioneers groundbreaking therapeutic avenues for neuroinflammation-targeted treatments in neurodegenerative disorders, including Alzheimer’s disease and Parkinson’s disease. Future investigations should prioritize the clinical translation of this nanodrug platform with an emphasis on optimizing personalized precision dosing regimens, ultimately aiming to accomplish the dual therapeutic objectives of enhanced radiotherapy efficacy and concomitant neuroprotection.

## Materials and methods

### Cell culture

Murine microglia (BV2), hippocampal neuronal cell line (HT22) and mouse glioma cell line (GL261-luc) were obtained from Shanghai Saibaikang Biotechnology Co., Ltd. (Shanghai, China). Both cell lines were routinely cultured in high-glucose Dulbecco’s modified Eagle’s medium (DMEM, Gibco) supplemented with 10% fetal bovine serum (FBS, Gibco) and 1% penicillin‒streptomycin solution (containing 100 U/mL penicillin and 100 μg/mL streptomycin; HyClone). The cell cultures were maintained in a humidified incubator at 37 °C with a 5% CO_2_ atmosphere, with regular medium changes and subculturing performed according to standard cell culture protocols.

### Cell irradiation treatment and conditioned medium transfer

BV2 cells were seeded in six-well plates at a density of 5 × 10⁴ cells/cm² and irradiated with a ^137^Cs γ-ray source (0.88 Gy/min), with doses adjusted according to experimental protocols. After irradiation, the cultures were maintained for an additional 24 h, after which the supernatants were collected and subjected to sequential processing. For the in vitro co-culture model, HT22 cells were plated in 24-well plates at 3 × 10⁴ cells/cm². Upon reaching 60% confluency, the culture medium was replaced with conditioned medium containing 50% (v/v) irradiated BV2-derived supernatant. The cells were incubated under these co-culture conditions for 24 h before subsequent analyses.

### Zebrafish husbandry and irradiation

Adult zebrafish (*Danio rerio*, AB strain) were provided by Naniing EzeRinka Biotechnology Co., Ltd., and maintained in a recirculating aquaculture system (Shanghai Haisheng Biotech Company, China) under standardized conditions: water temperature 28.5 ± 0.5 °C, pH 7.0–7.5, conductivity 500–1500 μS/cm, and dissolved oxygen >6 mg/L, with a 14:10 h light–dark photoperiod. All procedures were approved by the Animal Care Committee and authorized by the Ethics Committee of the Institute of Radiation Medicine of the Peking Union Medical College (Approval ID: IRM2-IACUC-2507-014).

For embryo production, sexually mature breeders were segregated overnight in spawning tanks with partitions at a 1:1 female-to-male ratio. Partitions were removed 30 min after light initiation, allowing spontaneous spawning within 60 min. Embryos were collected through 300 μm nylon mesh and washed thrice with E3 media (5 mM NaCl, 0.17 mM KCl, 0.33 mM CaCl₂, 0.33 mM MgSO₄) containing 0.003% methylene blue to eliminate debris and unfertilized eggs.

Embryos at 6 hpf were subjected to whole-body irradiation with a single dose of 6 Gy, delivered at a dose rate of 0.84 Gy/min for a total duration of 7 min. Following irradiation, the embryos were placed in 24-well plates (20 embryos per well) and maintained at 28.5 °C. The E3 medium was changed daily, and the embryos were monitored for developmental abnormalities.

Six-month-old zebrafish (body weight: 0.3–0.5 g) underwent whole-body irradiation with a single dose of 20 Gy, which was administered at a dose rate of 0.84 Gy/min for a total duration of 24 min. Postirradiation, the fish were transferred to an individual recirculating water system and monitored continuously for behavioral alterations and feeding activity.

### Murine husbandry and irradiation

Eight-week-old male C57BL/6J mice (Beijing Huafukang Biotechnology Co., Ltd., China) were housed under specific pathogen-free (SPF) conditions (temperature: 22 ± 1 °C, relative humidity: 50 ± 10%, 12h light/dark cycle). All experimental protocols were approved by the Animal Care Committee and authorized by the Ethics Committee of the Institute of Radiation Medicine of the Peking Union Medical College (Approval ID: IRM2-IACUC-2507-012). Prior to irradiation, the mice were anesthetized via intraperitoneal injection of 2% pentobarbital sodium, with irradiation commencing upon confirmed loss of nociceptive reflexes. Localized irradiation was performed via an X-RAD 320ix precision irradiator equipped with a custom lead collimator (2 × 2 cm² exposure field). The operating parameters included a 160 kV tube voltage, a 25 mA current, and a dose rate of 1.8 Gy/min. The cells were irradiated continuously for 5 days at a dose of 5 Gy each, and the cumulative total irradiation dose was 25 Gy.

### Spontaneous embryonic contraction assay

Twenty-four hpf zebrafish embryos were individually placed in six-well plates (20 embryos/well containing 5 mL of fresh E3 medium) for locomotor assessment. Embryonic movements were continuously recorded for 1 min under a stereomicroscope (SMZ1270, Nikon, Japan). Tail coiling events, defined as periodic contractions with ≥90° caudal flexion amplitude, were quantified via Danio Scope software (Noldus, Netherlands). The mean contraction frequency (contractions/min/embryo) was calculated to assess neuromuscular development.

### Zebrafish swimming behavioral assessment

Larvae (120 hpf) were individually transferred to circular 96-well behavioral chambers. Following 10 min of environmental acclimation, spontaneous locomotion was video recorded for 10 min, after which the samples were subjected to cyclic light‒dark stimulation (10 min light/10 min dark, 3 cycles) in 96-well chambers after acclimation. Acute locomotor response intensity was specifically analyzed during the 1 min window following light‒to‒dark transitions via EthoVision XT 15 software (Noldus, Netherlands).

### Y-Maze spontaneous alternation test

The Y-maze apparatus was composed of three identical, gray, opaque acrylic arms. Under standardized experimental conditions, the exploratory behavior of the test subjects was captured by an infrared video tracking system.^60^ After 24 h of pretest habituation, the mice underwent a 10-min free exploration trial initiated from a designated start arm.

### Novel object recognition paradigm

The mice were acclimated to an empty open-field arena (40 × 40 × 40 cm^3^) through daily 10-min exposures for three consecutive days. During the training phase, two identical metallic spheres (5 cm diameter, 10 cm height) were fixed diagonally in the arena. For formal testing, exploratory interactions were video recorded for 10 min via an ANY-maze tracking system. One sphere was replaced with a novel cube of equivalent volume during the test phase.

### Acridine orange (AO) staining of zebrafish embryos

At 120 hpf, the larvae were immersed in 5 μg/mL AO solution (0.1% DMSO in E3 medium; Sigma-Aldrich) under light-protected conditions with agitated incubation (37 °C, 30 min). Following triple washing in fresh E3 medium and fixation with 4% PFA (4 °C, 24 h), apoptotic cells were visualized via a Nikon SMZ1270 fluorescence microscope (excitation/emission: 490/530 nm; GFP filter set). Quantitative analysis of the density of AO-positive foci was performed via ImageJ (v1.53) with automated thresholding and region-of-interest (ROI) selection.

### In vitro reactive oxygen species (ROS) detection

BV2 microglia were incubated with 10 μM 2’,7’-dichlorodihydrofluorescein diacetate (DCFH-DA, Solarbio), a cell-permeable ROS-sensitive fluorogenic probe, under light-protected conditions (37 °C, 30 min) prior to irradiation/pharmacological treatments. The extracellular probes were removed by washing with PBS after incubation, and nuclear counterstaining with DAPI (5 μg/mL, 10 min) was performed before confocal microscopy analysis (Leica, 7CSSP8; 488 nm excitation/505 nm emission) for qualitative ROS visualization.

For quantitative assessment, single-cell suspensions were analyzed via flow cytometry (BD FACSCanto™ II) with 488 nm excitation and 530/30 nm emission filters. A minimum of 10,000 singlet-gated live cells per sample were acquired, and the mean fluorescence intensity (MFI) of DCFH-DA was quantified via FlowJo v10.8. Data normalization incorporated unstained controls and vehicle-treated samples to account for autofluorescence and nonspecific signals.

### In vitro detection of mitochondrial reactive oxygen species (mtROS)

BV2 microglia were seeded in 24-well plates. Prior to irradiation, the cells were pretreated with ROS scavengers (NAC or MitoTEMPO) and nanotherapeutics. At 6 h post-irradiation, the cells were costained with 2 μM MitoSOX™ Red (for mitochondrial ROS detection, Beyotime) and MitoTracker™ Green (for mitochondrial localization, Beyotime) in serum-free medium at 37 °C for 10 min under light-protected conditions. Following three washes with serum-free medium, mtROS levels were quantified by measuring red fluorescence (Ex/Em = 510/580 nm) via confocal microscopy, and mitochondrial localization was confirmed via 488 nm laser excitation of MitoTracker Green.

### TEM analysis of mitochondrial ultrastructure

*Sample preparation*: BV2 cells in the logarithmic growth phase were trypsinized and pelleted via centrifugation (1000 rpm, 5 min). The cell pellets were fixed with prechilled 2.5% glutaraldehyde (in 0.1 M phosphate buffer, pH 7.4) at 4 °C for 2 h, followed by three 10-min washes with 0.1 M phosphate buffer (pH 7.4). Postfixation was performed via the addition of 1% osmium tetroxide (OsO₄) at 4 °C for 1.5 h.

*Dehydration and embedding*: Samples were dehydrated through a graded ethanol series (50%, 70%, 90%, and 100%, 15 min each), transitioned with propylene oxide, and infiltrated with epoxy resin (Epon 812). Polymerization was conducted at 60 °C for 48 h.

*Ultrathin sectioning and staining*: Resin-embedded blocks were sectioned into 70 nm slices via an ultramicrotome (Leica UC7). The sections were mounted on copper grids and double-stained with 2% uranyl acetate (15 min) and lead citrate (15 min) at room temperature.

*TEM imaging*: Stained sections were analyzed under a transmission electron microscope (TEM, Hitachi HT7800) operated at 120 kV. The mitochondrial ultrastructure (cristal morphology, matrix density, and outer membrane integrity) was visualized in high-angle annular dark-field (HAADF) mode.

### Live-cell mitochondrial imaging and mtDNA localization

The cells were dual-labeled with 200 nM PK mito Deep Red (Genvivotech, PKMDR-1) and SYBR™ Green (1:1000 dilution; Invitrogen, S11494) in serum-free medium under light-protected conditions (37 °C, 30 min). Mitochondrial architecture and mtDNA spatial distribution data were acquired on a Multi-SIM X (Multimodality Structured Illumination Microscopy X) imaging system (NanoInsights-Tech Co., Ltd.) equipped with a ×63 1.46 NA oil objective. Sequential excitation at 488 nm (SYBR Green I; Ex/Em: 497/520 nm) and 640 nm (PK mito Deep Red; Ex/Em: 644/670 nm) minimized the spectral overlap, with z-stack acquisition (0.2 μm intervals) for 3D reconstruction. Mitochondrial network analysis was performed via ImageJ v1.53 with the MiNA macro suite. Morphometric parameters, including the mitochondrial footprint (area occupied by mitochondria), network branch length, and junction density, were quantified following adaptive thresholding and skeletonization algorithms.

#### Western blot

Protein extracts were obtained via RIPA lysis buffer (containing 1× protease/phosphatase inhibitor cocktail) and quantified via a BCA assay (Solarbio). Equal amounts of protein (20–50 μg/lane) were resolved on 8–12% gradient SDS‒polyacrylamide gels under reducing conditions and transferred to preactivated PVDF membranes (0.45 μm, Millipore) via transfer. The membranes were blocked with 5% nonfat dry milk in TBST (Tris-buffered saline with 0.1% Tween-20) for 1 h at room temperature, followed by sequential incubation with primary antibodies (listed in Supplementary Table 1) at 4 °C overnight and HRP-conjugated secondary antibodies (anti-rabbit/mouse IgG, 1:5000; room temperature, 1 h). For signal development, an enhanced chemiluminescence substrate (ECL Prime, Biosharp) was used, and images were acquired via a ChemiDoc™ MP Imaging System (Bio-Rad). Band intensity quantification was performed via Image Lab™ software (v6.1), with normalization to the level of the GAPDH loading control.

#### Quantitative real-time PCR analysis

Total RNA was isolated from cells, zebrafish embryos and murine brain tissues via TRIzol® Reagent (Takara), followed by DNase I treatment to eliminate genomic DNA contamination. The RNA purity and concentration were verified via a NanoDrop 2000 spectrophotometer (Thermo). Reverse transcription was performed with 1 μg of RNA via PrimeScript™ RT Master Mix (Yeason) under optimized thermal conditions (37 °C for 15 min; 85 °C for 5 s inactivation).

For the mitochondrial DNA quantification assay, BV2 microglia were seeded in six-well plates. Following irradiation/drug treatment, the cells were washed twice with ice-cold PBS. Cytosolic fractions were isolated via gently pipetting freshly prepared cytosolic extraction buffer (10 mM Tris–HCl pH 7.4, 10 mM NaCl, 3 mM MgCl₂, 0.1% Triton X-100). After 5 min of incubation on ice, the samples were centrifuged at 12,000 rpm for 30 min at 4 °C. The supernatant (cytosolic fraction) was transferred to new tubes, and the pellets (nuclear fraction) were discarded. Cytosolic DNA was extracted via a FastPure Cell DNA isolation kit (Vazyme). Quality control was performed by quantifying nuclear gene GAPDH contamination. Cytosolic fractions with a GAPDH Ct > 32 cycles were considered free of significant nuclear DNA contamination. The mtDNA copy number was normalized to that of 18S rDNA as an endogenous control.

The qPCRs (20 μL final volume) included SYBR Green® Premix Ex Taq II (Roche), 10 μM gene-specific primers (sequences listed in Supplementary Table 2), and diluted cDNA (1:10). The amplification parameters included initial denaturation (95 °C, 30 s), 40 cycles of two-step amplification (95 °C for 5 s, 60 °C for 30 s), and melt curve analysis (60–95 °C, 0.5 °C/s increments). All reactions were performed in triplicate with no-template controls. Relative gene expression was calculated via the ^ΔΔ^Ct method and normalized to that of β-actin or rsp18. Statistical significance was determined via one-way ANOVA with Tukey’s post hoc test in GraphPad Prism 9.0.

### Histological staining

Zebrafish larvae, adult zebrafish and murine tissues were fixed in 4% paraformaldehyde (PFA; Sigma-Aldrich) for 24 h at 4 °C. Following fixation, all the samples were subjected to sequential ethanol dehydration (70–100% gradient), xylene clearance, and paraffin embedding. Serial sections were prepared via a rotary microtome. HE-stained dewaxed sections were processed as previously described. Briefly, the sections were stained with Harris-modified hematoxylin (Solarbio) for 5 min, differentiated in 1% acid ethanol (10 s), and rinsed under running water for 20 min to restore basophilic staining. Counterstaining with 0.5% eosin Y (Sigma) was performed for 1 min, followed by gradient dehydration and mounting with neutral resin. Nissl staining was conducted via an optimized toluidine blue protocol. Deparaffinized sections were incubated in 0.1% toluidine blue (pH 4.7, 60 °C) for 10 min, rapidly differentiated in 95% ethanol‒xylene, and mounted for microscopic visualization of Nissl bodies. High-resolution whole-slide imaging was performed using a Hamamatsu NanoZoomer S60 digital scanner.

Golgi-Cox impregnation was performed according to previously described methods. Fresh murine brains were immersed in prechilled Golgi-Cox solution (5% potassium dichromate/5% mercuric chloride/5% potassium nitrate, 1:1:1 v/v) under light-protected conditions for 14 days, followed by dehydration with 30% sucrose (3 days, 4 °C). Sagittal sections (100 μm) were prepared via a vibratome (Leica VT1200S), silver-enhanced, and DPX-mounted. Neuronal dendritic complexity was analyzed from NanoZoomer S60-scanned images.

### Immunofluorescence staining

All immunostaining procedures followed standardized protocols. The cell monolayers were fixed with 4% paraformaldehyde (PFA) and permeabilized with 0.1% Triton X-100, while the paraffin sections were deparaffinized, followed by 20 min of antigen retrieval in citrate buffer (pH 6.0, 95 °C). After being blocked with 5% bovine serum albumin (BSA), the samples were incubated overnight at 4 °C with primary antibodies targeting key markers: iNOS (M1 macrophage), TNF-α (proinflammatory cytokine), γ-H2AX (DNA damage), CD86/CD206 (M1/M2 polarization), NeuN (neuronal nuclei), p-STING (activated STING pathway), Iba1 (microglial activation), and p-NF-κB (inflammatory signaling) (listed in Supplementary Table 1). Corresponding species-matched secondary antibodies conjugated to Alexa Fluor® 488/594 (1:1000 dilution; Thermo Fisher Scientific) were applied for 1 h at room temperature, with DAPI counterstaining (5 min) for nuclear visualization. Confocal imaging was performed via a Zeiss LSM900 system, and quantitative fluorescence analysis was performed via ImageJ (v1.53) with automated threshold segmentation and region-of-interest (ROI) quantification protocols to determine the relative intensity of the target signals.

### Immunohistochemical staining

Paraffin-embedded murine brain sections were subjected to deparaffinization through sequential xylene immersion (3 × 10 min) and rehydration with graded ethanol (100–70%), followed by antigen retrieval in preheated citrate buffer (10 mM, pH 6.0) via microwave-mediated heating (95 °C, 15 min). After the samples were cooled to room temperature and rinsed with PBS, endogenous peroxidase activity was quenched with 3% H₂O₂/methanol (15 min, dark), and nonspecific binding was blocked with 5% normal goat serum containing 0.3% Triton X-100 (1 h, 37 °C). The sections were incubated overnight at 4 °C with primary antibodies (listed in Supplementary Table 1), followed by incubation with HRP-conjugated secondary antibodies (1:1000; Dako #K4003; 1 h, RT) and DAB chromogenic detection (Sigma #D3939) under microscopic monitoring. Nuclei were counterstained with hematoxylin, differentiated in acid alcohol, dehydrated, cleared in xylene, and mounted with neutral resin. Digital images were acquired via an Olympus BX53 microscope, and positive staining areas (%) in target regions were quantified across five randomized fields per sample via Image-Pro Plus 6.0, with statistical significance (**p < 0.01) determined via Student’s *t*-test (GraphPad Prism 9.0).

### GL261 orthotopic glioma model in mice

C57BL/6 female mice (6–8 weeks old, 18–22 g) were used for modeling, with all procedures approved by the Institutional Animal Care and Use Committee. GL261-luc mouse glioblastoma cells were cultured in high-glucose DMEM supplemented with 10% fetal bovine serum and 1% penicillin‒streptomycin at 37 °C in a 5% CO₂ incubator. For cell preparation, confluent cells were trypsinized (0.25% trypsin), centrifuged at 1000 rpm for 5 min, washed twice with PBS, and resuspended in PBS at a concentration of 1 × 10^6^ cells/mL. The cell suspension was mixed 1:1 with Matrigel to achieve a final concentration of 5 × 10⁵ cells/mL and kept on ice until use.

The mice were anesthetized with isoflurane (4% induction, 2% maintenance) and fixed in a stereotactic frame (Stoelting SR-5 N). After scalp disinfection and midline incision, the skull was exposed, and the bregma served as the coordinate origin. A burr hole (0.5 mm diameter) was drilled 2.0 mm posterior and 2.5 mm lateral to the bregma, and a 10-μL microsyringe was inserted 3.5 mm deep into the brain parenchyma. A 5-μL cell–Matrigel mixture (2.5 × 10³ cells) was injected at a rate of 1 μL/min, followed by a 5-min dwell time before slow needle withdrawal. The burr hole was sealed with bone wax, and the scalp was sutured. Postoperatively, the body weights and neurological symptoms of the mice were monitored daily. Tumor growth was evaluated by in vivo fluorescence imaging using D-Luciferin potassium salt post-injection and irradiation.

### In vivo fluorescence imaging in murine models

Seven days following localized cranial χ-ray irradiation, the mice received intravenous tail vein administration of Cy5-conjugated material (a specific formulation to be detailed). After cranial hair removal, longitudinal fluorescence tracking was performed via an IVIS® Lumina III imaging system (PerkinElmer). Anesthesia was induced with a 2% isoflurane/oxygen mixture (1 L/min flow rate) and maintained via a 37 °C thermoregulated stage in the prone position. Dynamic signal acquisition employs autoexposure optimization (1–60 s) with spectral unmatching filters (Ex/Em: 640/680 nm).

### Synthesis of Pep-Cu_5.4_O@H151

*Synthesis of the Cu*_5.4_*O nanozyme*: First, 10 mM CuCl_2_ powder was dissolved in 50 mL of deionized water, and the solution was stirred at 80 °C in an oil bath for 10 min. Then, 100 mM ascorbic acid was slowly added to the CuCl_2_ solution, and the pH was adjusted to a weakly alkaline value (~pH 8.5) with 1 M NaOH. The reaction was carried out in an oil bath at 80 °C for 12 h. The large particles were removed by centrifugation at 6500 × *g* for 5 min. The product was then dialyzed and freeze-dried to obtain the Cu_5.4_O nanozyme, which was quantitatively analyzed via ICP‒MS.

*Synthesis of the Pep-Cu*_5.4_*O nanozyme*: Five milligrams of peptide were dissolved in 10 mL of deionized water. Under high-speed stirring conditions, different concentrations of the Cu_5.4_O nanozyme were gradually added dropwise. During addition, Pep-Cu_5.4_O nanozymes formed rapidly. The mixture was stirred for an additional 4 h to allow the Pep-Cu_5.4_O nanozyme to gradually stabilize. Finally, the Pep-Cu_5.4_O nanozyme was recovered via centrifugation, and quantitative analysis was performed via ICP‒MS.

*Drug loading efficiency of the Pep-Cu*_5.4_*O nanozyme*: Different amounts of the drug H151 were dissolved in acetone, and then the H151 solution was added to the stirred Pep-Cu_5.4_O nanozyme solution for drug loading. During this process, organic solvents are removed via methods such as heating and vacuum pumping, allowing H151 to be retained within the Pep-Cu_5.4_O nanozyme. The unloaded drug was removed by repeated centrifugation. The absorbance of the supernatant was measured, and the drug loading efficiency of the Pep-Cu_5.4_O nanozyme was calculated on the basis of a fitted standard concentration curve of H151.

*Drug release profile of Pep-Cu*_5.4_*O@H151*: Pep-Cu_5.4_O@H151 was dispersed in deionized water and placed into a dialysis bag with a molecular weight cutoff (MWCO) of 3.5 kDa. The bag was sealed and placed into a conical flask, and 49 mL of release medium was added to simulate in vitro release. The release experiment was conducted at 37 °C with continuous shaking at 120 rpm. At specific time points (0, 1, 2, 4, 6, 8, 12, 24, and 48 h), 1 mL of solution was collected from the conical flask, and an equal volume of fresh release medium was added to maintain the volume. After all the samples were collected, the concentration of H151 was quantified via a UV‒Vis spectrophotometer, and the cumulative release of H151 at each time point was calculated.

### RONS scavenging

*Hydrogen peroxide (H*_2_*O*_2_*) clearance*: H_2_O_2_ (0.1 mM) was incubated with Pep-Cu_5.4_O@H151 for 3 h to catalyze the decomposition of H_2_O_2_. The residual H_2_O_2_ in the solution is subsequently detected via a cerium sulfate (Ce(SO_4_)_2_) redox method. In this process, H_2_O_2_ reacts with tetravalent cerium (Ce(IV)), reducing cerium sulfate to trivalent cerium (Ce(III)). The UV absorbance of Ce(III) at 319 nm was measured via a UV‒Vis spectrophotometer to quantify the remaining H_2_O_2_.

*Superoxide anion (O*^2−^*) clearance*: Under weakly alkaline conditions, pyrogallol undergoes autoxidation to generate a superoxide anion (O^2−^) and a colored intermediate product, which has a characteristic absorption peak at 320 nm. In preliminary experiments, the amount of intermediate product generated was linearly related to the reaction time. After the addition of the Pep-Cu_5.4_O@H151 nanozyme, it rapidly reacts with O^2−^, preventing the accumulation of the intermediate product and reducing the absorbance at 320 nm. The O^2−^ scavenging efficiency of the Pep-Cu_5.4_O@H151 nanozyme was evaluated by measuring the 320 nm value.

*Hydroxyl radical (·OH) clearance*: H_2_O_2_ reacts with ferrous ions to generate ·OH, which has a short lifespan and high reactivity. When salicylic acid is added to the reaction system, it quickly captures ·OH to form a purple compound (2,3-dihydroxybenzoic acid), which has a significant absorption peak at 510 nm. The absorbance at 510 nm is proportional to the amount of ·OH. After the addition of the Pep-Cu_5.4_O@H151 nanozyme, the amount of oxidized salicylic acid decreased, leading to a reduction in the system’s color intensity or even its complete disappearance. The decrease in absorbance reflects the ability of the nanozyme to scavenge hydroxyl radicals.

### ABTS decolorization assay for total antioxidant capacities

The total antioxidant capacity (T-AOC) of all the Pep-Cu_5.4_O@H151 samples was determined via the ABTS chromogenic method with a T-AOC assay kit. The ABTS working solution was prepared by reacting equal volumes of ABTS and an oxidizing agent to generate ABTS•^+^ radical cations, which were allowed to develop over 16 h. Different concentrations of Pep-Cu_5.4_O@H151 were added to the ABTS working solution and allowed to react for 25 min. The decrease in absorbance at 734 nm, corresponding to the inhibition of ABTS•^+^ generation by the samples, was measured. Trolox was used as the reference antioxidant standard to quantify the results.

### Statistical analysis and software

The data were analyzed via appropriate methods and are presented as the means ± SEMs with respect to the number of samples (*n*) in each group. Statistical comparisons between two groups were performed via Student’s *t*-test, whereas comparisons among more than two groups were conducted via one-way analysis of variance (ANOVA) via GraphPad Prism software. A *p*-value of *p < 0.05, **p < 0.01, and ***p < 0.001 was considered statistically significant. The quantification of fluorescence and the immunohistochemical analysis were performed via ImageJ software (version 10.3.1). The scheme and illustrations were derived from BioRender (https://app.biorender.com). Peptide sequences were drawn using ChemDraw software (version 22).

## Supplementary information

Uncropped Western blot pictures

Supplementary Materials

Movie S1

Movie S2

## Data Availability

All the data generated or analyzed during this study are included in this published article and its supplementary information files.
