# Supplementary material for: Understanding the toxicity induced by radiation-triggered neuroinflammation and the on-demand design of targeted peptide nanodrugs
Source: Signal Transduct Target Ther. 2025 Sep 4;10:286. doi: 10.1038/s41392-025-02375-9 (PMC12408838; doi:10.1038/s41392-025-02375-9)
Supplement: Supplementary file 1 — Uncropped Western blot pictures [file 41392_2025_2375_MOESM1_ESM.ppt]

## Slide 1
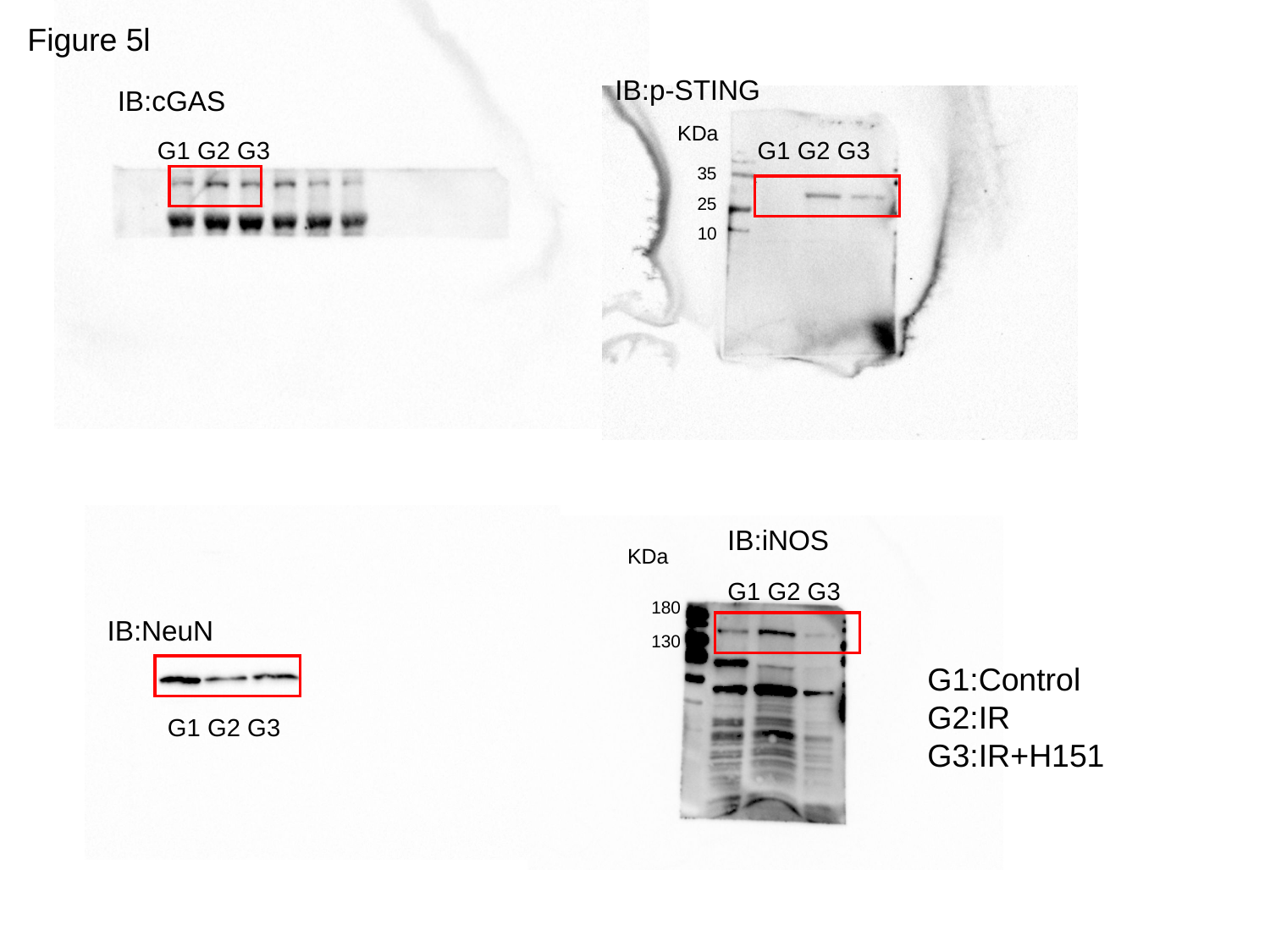

Figure 5l
IB:p-STING
IB:cGAS
KDa
G1 G2 G3
G1 G2 G3
35
25
10
IB:iNOS
KDa
G1 G2 G3
180
IB:NeuN
130
G1:Control
G2:IR
G3:IR+H151
G1 G2 G3

## Slide 2
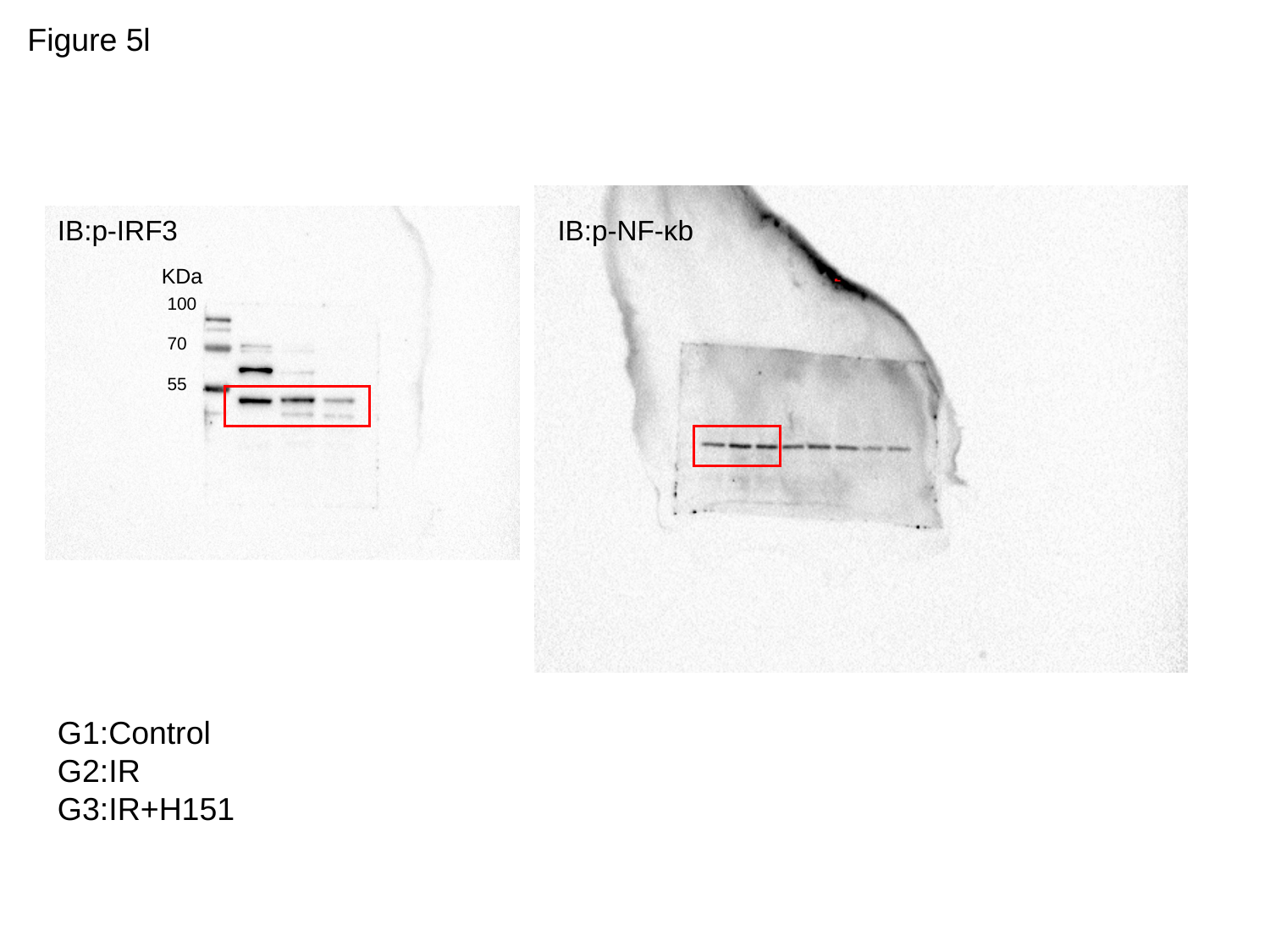

Figure 5l
IB:p-IRF3
IB:p-NF-κb
KDa
100
70
55
G1:Control
G2:IR
G3:IR+H151

## Slide 3
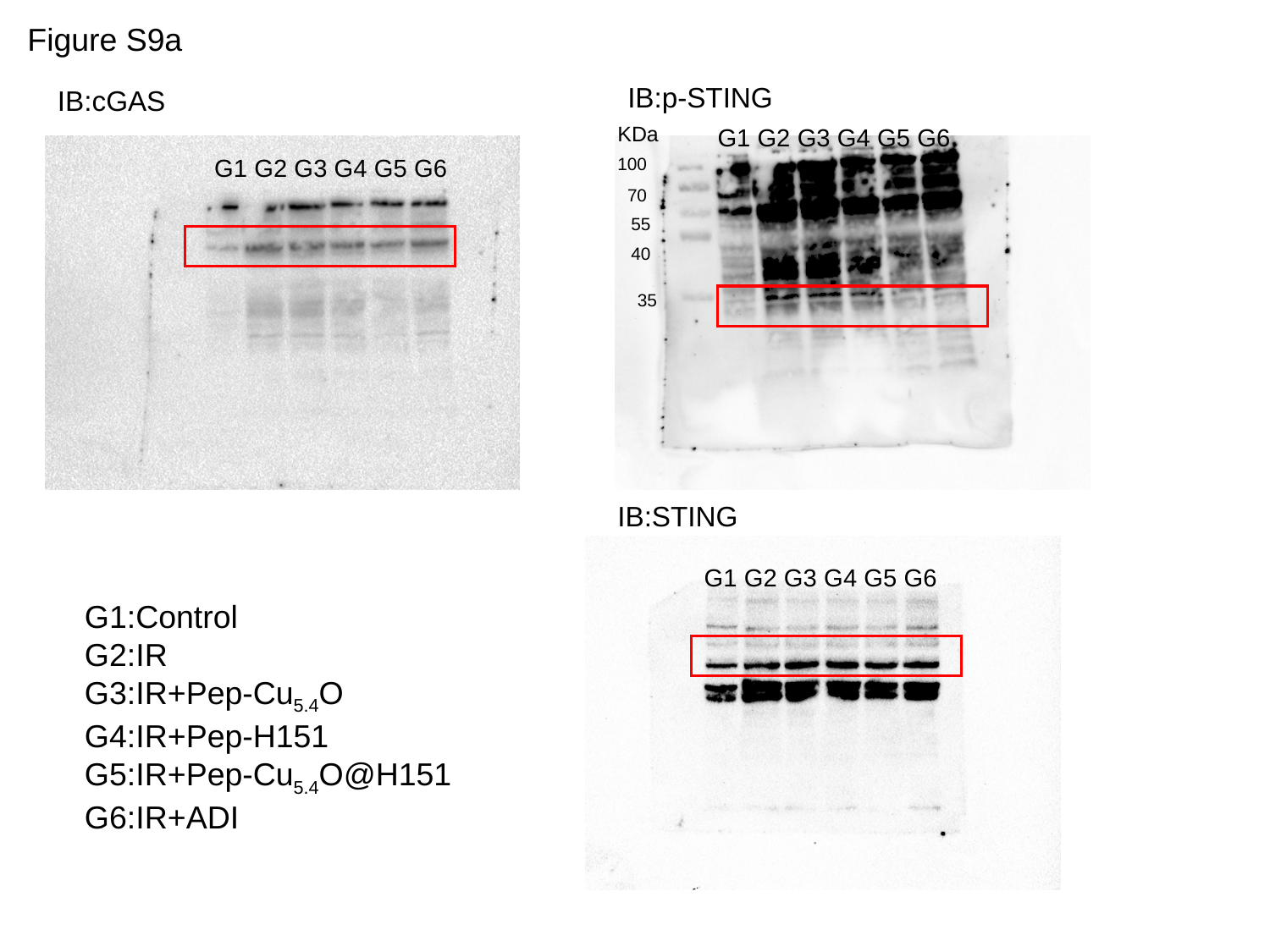

Figure S9a
IB:p-STING
IB:cGAS
KDa
G1 G2 G3 G4 G5 G6
G1 G2 G3 G4 G5 G6
100
70
55
40
35
IB:STING
G1 G2 G3 G4 G5 G6
G1:Control
G2:IR
G3:IR+Pep-Cu5.4O
G4:IR+Pep-H151
G5:IR+Pep-Cu5.4O@H151
G6:IR+ADI

## Slide 4
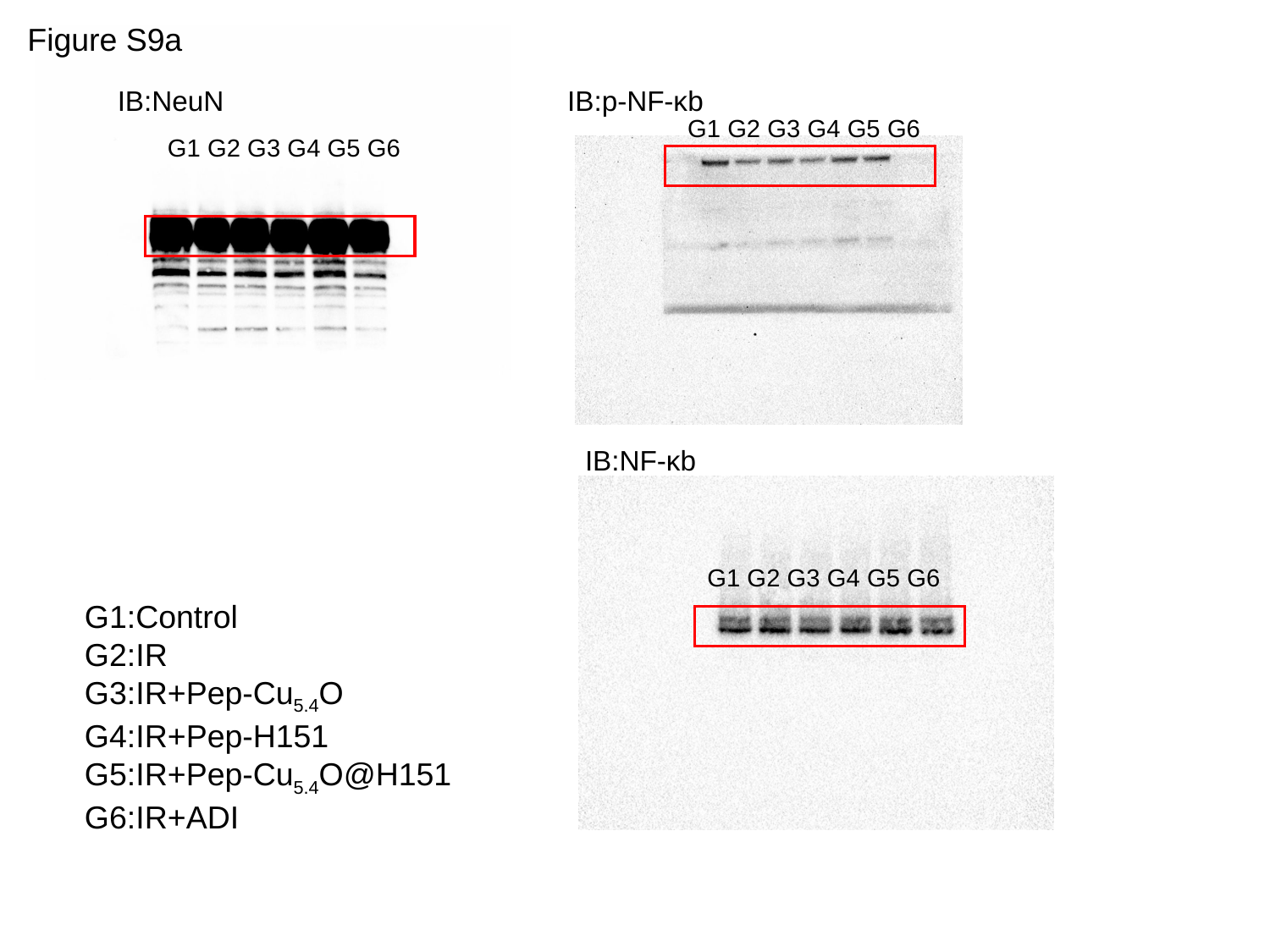

Figure S9a
IB:NeuN
IB:p-NF-κb
G1 G2 G3 G4 G5 G6
G1 G2 G3 G4 G5 G6
IB:NF-κb
G1 G2 G3 G4 G5 G6
G1:Control
G2:IR
G3:IR+Pep-Cu5.4O
G4:IR+Pep-H151
G5:IR+Pep-Cu5.4O@H151
G6:IR+ADI

## Slide 5
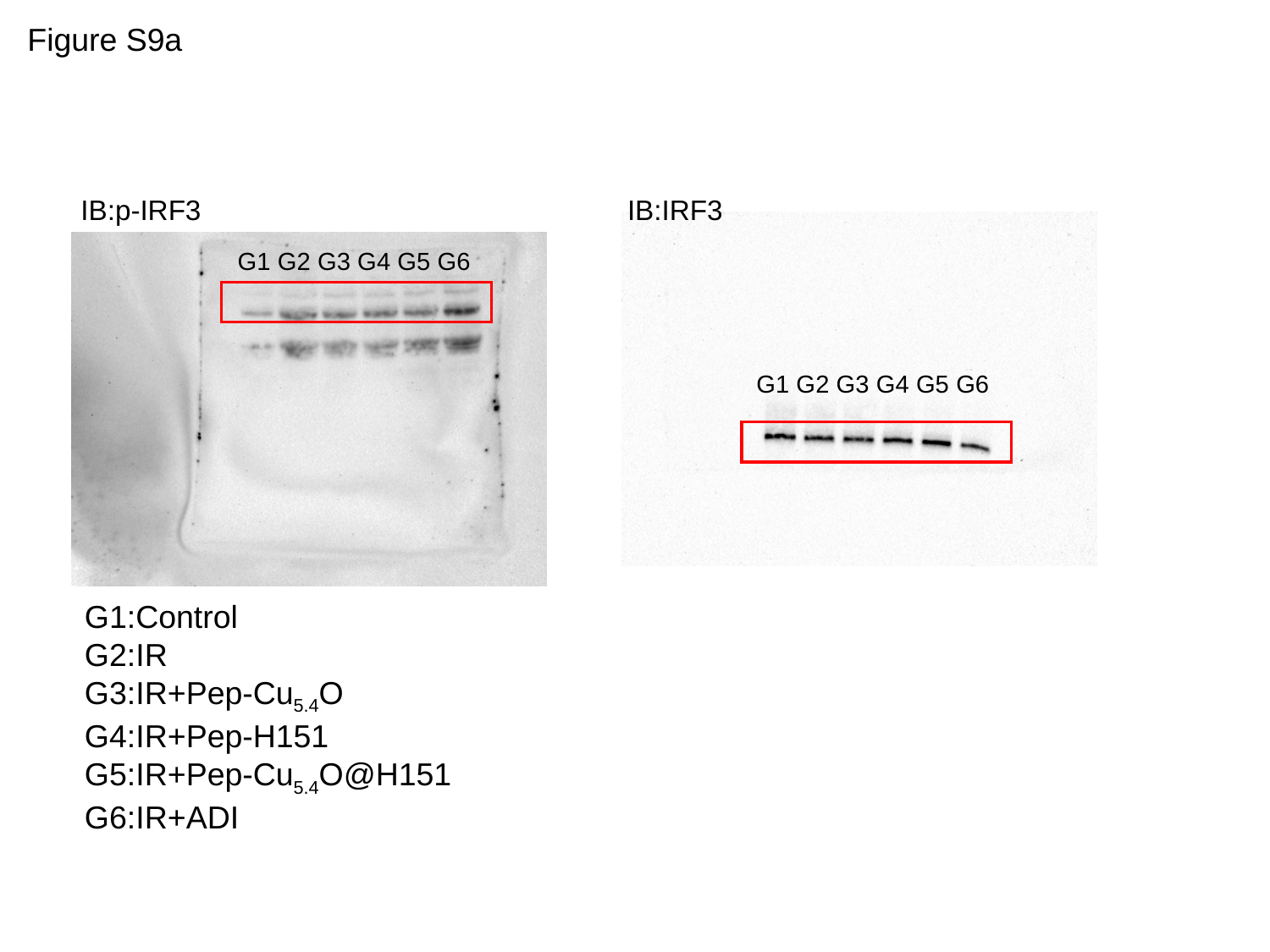

Figure S9a
IB:p-IRF3
IB:IRF3
G1 G2 G3 G4 G5 G6
G1 G2 G3 G4 G5 G6
G1:Control
G2:IR
G3:IR+Pep-Cu5.4O
G4:IR+Pep-H151
G5:IR+Pep-Cu5.4O@H151
G6:IR+ADI
