# Supplementary material for: Understanding the toxicity induced by radiation-triggered neuroinflammation and the on-demand design of targeted peptide nanodrugs
Source: Signal Transduct Target Ther. 2025 Sep 4;10:286. doi: 10.1038/s41392-025-02375-9 (PMC12408838; doi:10.1038/s41392-025-02375-9)
Supplement: Supplementary file 2 — Supplementary Materials [file 41392_2025_2375_MOESM2_ESM.docx]

Supplementary Materials for

**Understanding the Toxicity Induced by Radiation-Triggered Neuroinflammation and the On-Demand Design of Targeted Peptide Nanodrugs**

Yue Shang^1#^, Xueyin Hu^1#^, Meixia Ren^2#^, Longbo Ma^1^, Xiaoyu Zhao^1^, Cong Gao^1^, Lumeng Zhang^3^, Shuqin Li^1^, Luntao Liu^1^*, Bingwen Zou^4^*, Saijun Fan^1^*

Correspondence to: [fansaijun@irm-cams.ac.cn,](mailto:fansaijun@irm-cams.ac.cn) [zoubingwen81@163.com](mailto:zoubingwen81@163.com) and [liuluntao@irm-cams.ac.cn](mailto:liuluntao@irm-cams.ac.cn)

**This PDF file includes:**

Supplementary Figures. 1 to 9

Supplementary Tables. 1 to 2

**Other Supplementary Materials for this manuscript include the following:**

Supplementary Movies 1 to 2

Uncropped Western blot pictures


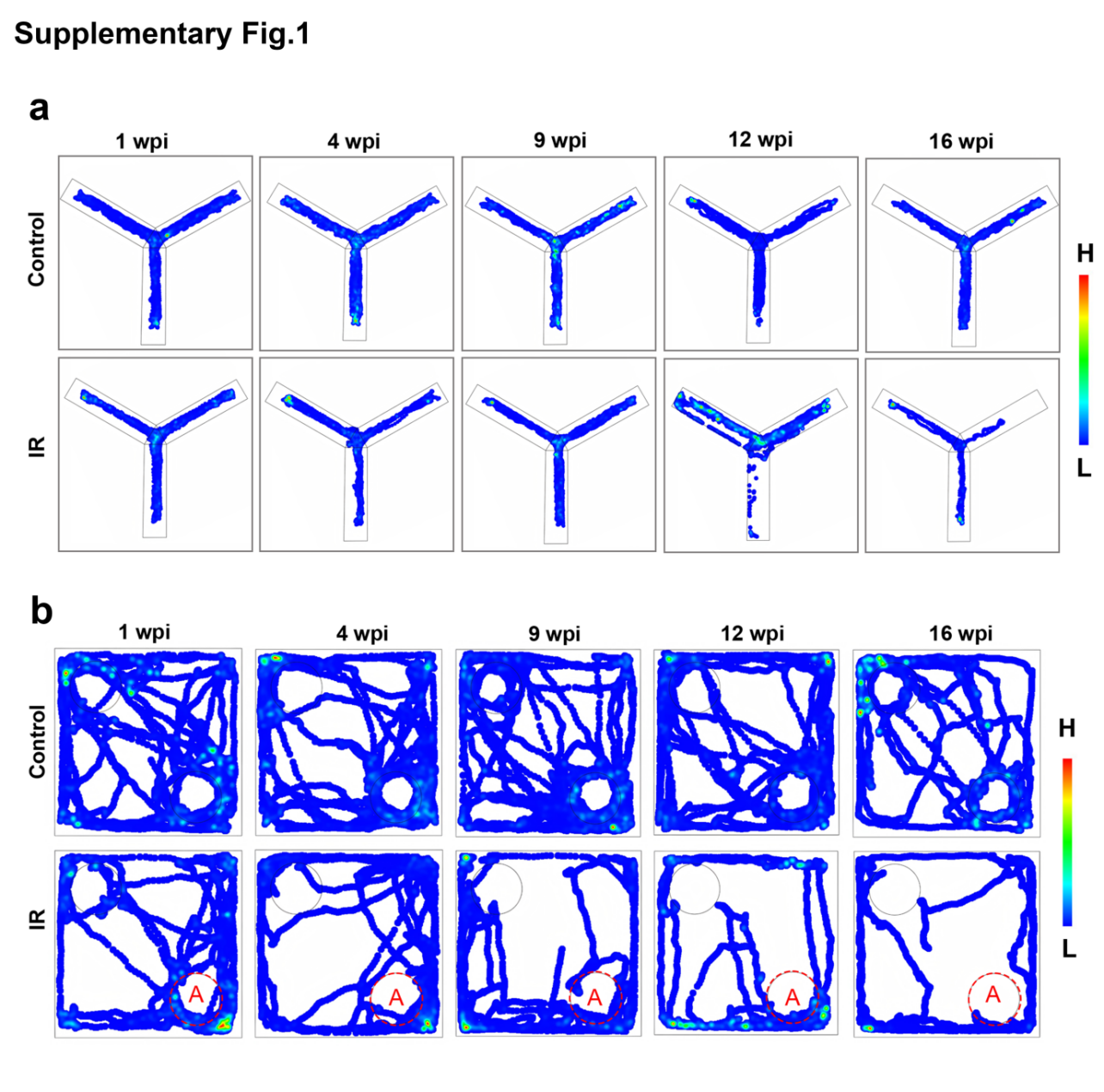


**Figure S1. (a)** Y-maze behavioral assay heatmaps illustrate exploration patterns at 1-, 4-, 9-, 12-, and 16-weeks post-cranial irradiation (wpi), with red hues indicating prolonged dwell time and blue tones reflecting baseline activity (red: high dwell time; blue: low). **(b)** NOR test heatmaps illustrate object (A) exploration patterns at 1-, 4-, 9-, 12-, and 16 wpi, with red hues representing increased novel object interaction and blue tones indicating baseline familiar object exploration.


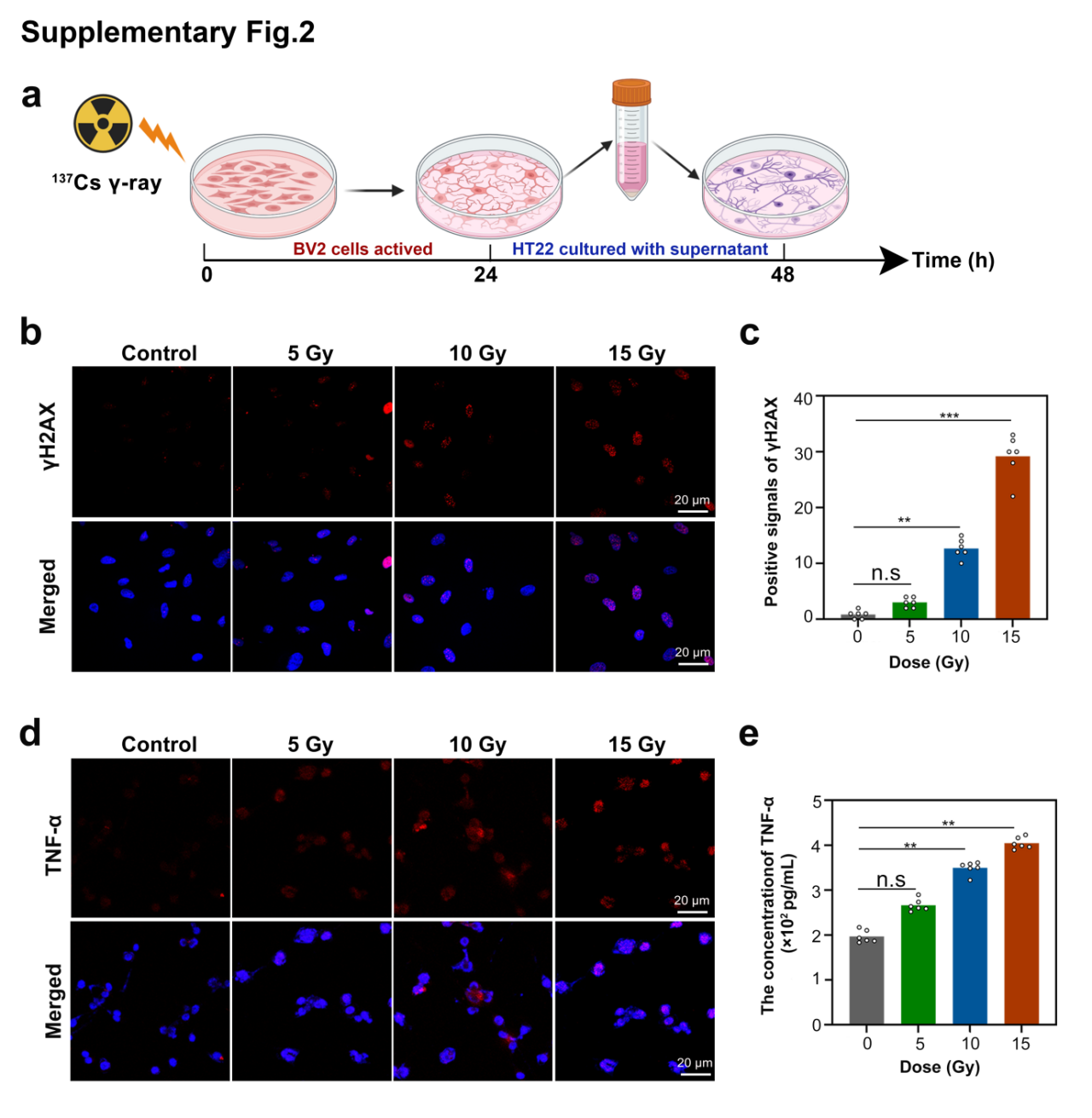


**Figure S2.** **(a)** Experimental timeline of irradiation-induced conditioned media transfer between microglia and hippocampal neurons. BV2 microglial cells were irradiated with γ-rays at graded doses (5, 10, 15 Gy ^137^Cs γ-ray)，culture media was collected at 24 hours post-irradiation and applied to HT22 hippocampal neurons for 24 h. **(b)** Representative γ-H2AX immunofluorescence staining in BV2 microglia 24 hours post-^137^Cs γ-irradiation (5, 10, and 15 Gy). **(c)** Quantification of γ-H2AX fluorescence intensity normalized to control. n = 3 biological replicates; ***P<0.001, **P<0.01; **Student’s *t*-test**. **(d)** TNF-α immunofluorescence staining in BV2 microglia 24 hours post-^137^Cs γ-irradiation (5, 10, and 15 Gy). **(e)** ELISA quantification of TNF-α concentration in culture supernatants. n = 3 biological replicates; **P<0.01; **Student’s *t*-test**. All the statistical data are presented as the means ± SEMs. Scale bar: 20 μm (b and d).

**
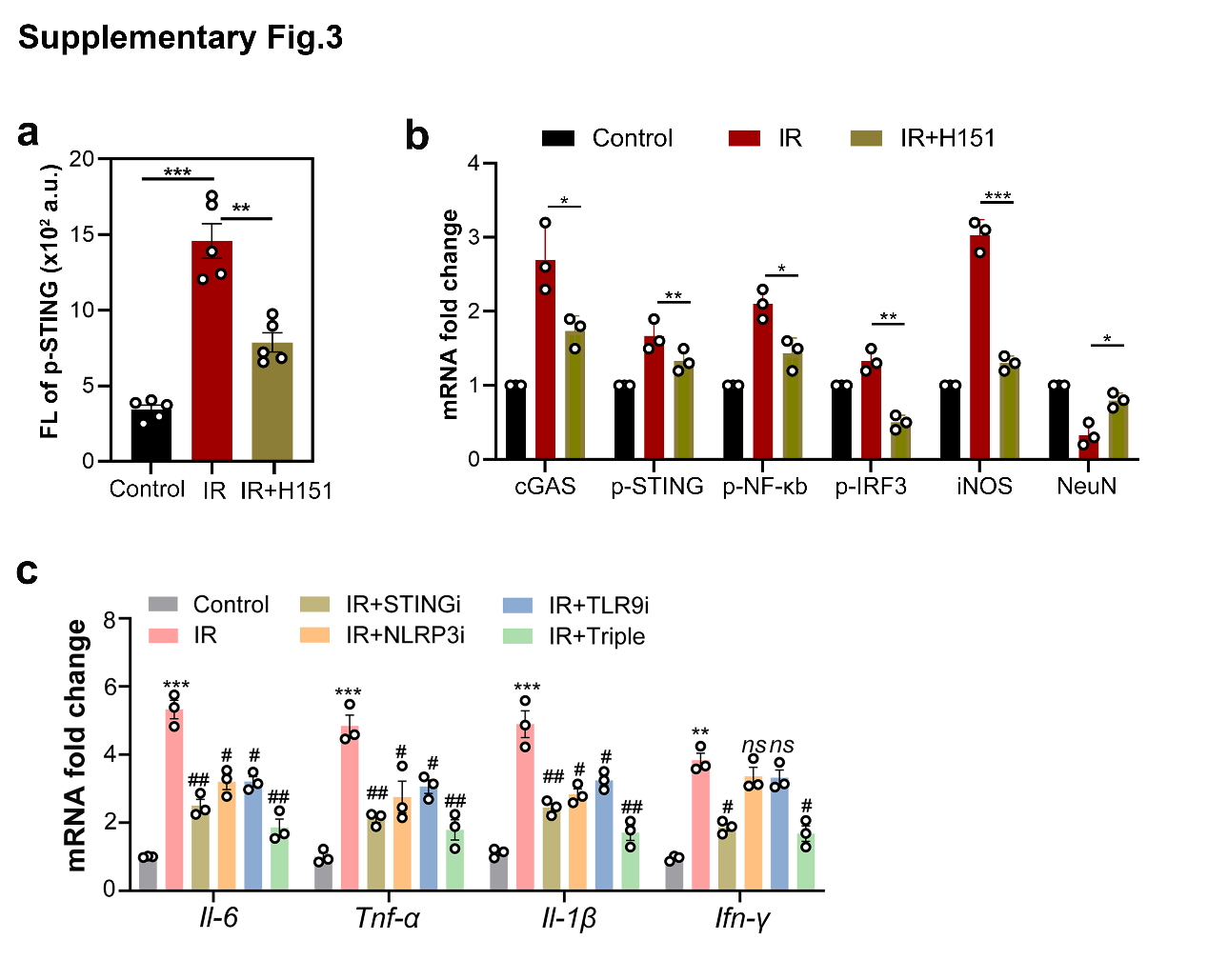
Figure S3. (a)** Fluorescence intensity analysis of p-STING. n = 3; **Student’s *t*-test**. **(b)** Quantitative analysis of protein expression levels. n = 3 biological replicates; **Student’s *t*-test**. **(c)** Inflammatory cytokine mRNA expression in BV2 microglia following individual and combinatorial inhibition of innate immune pathways. qPCR analysis of IL6, TNF-α, IL-1β, and IFN-γ mRNA levels after 24 h treatment with NLRP3 inhibitor: MCC950 (10 μM, NLRP3i), TLR9 inhibitor: ODN 2088 (5 μM, TLR9i) and STING inhibitor: H-151 (1 μM, STINGi). Data normalized to β-actin (n = 3 biological replicates; **p<0.01, ***p<0.001 vs. Control group; ^#^p<0.05, ^##^p<0.01 vs. IR group, two-way ANOVA). All the statistical data are presented as the means ± SEMs.

**
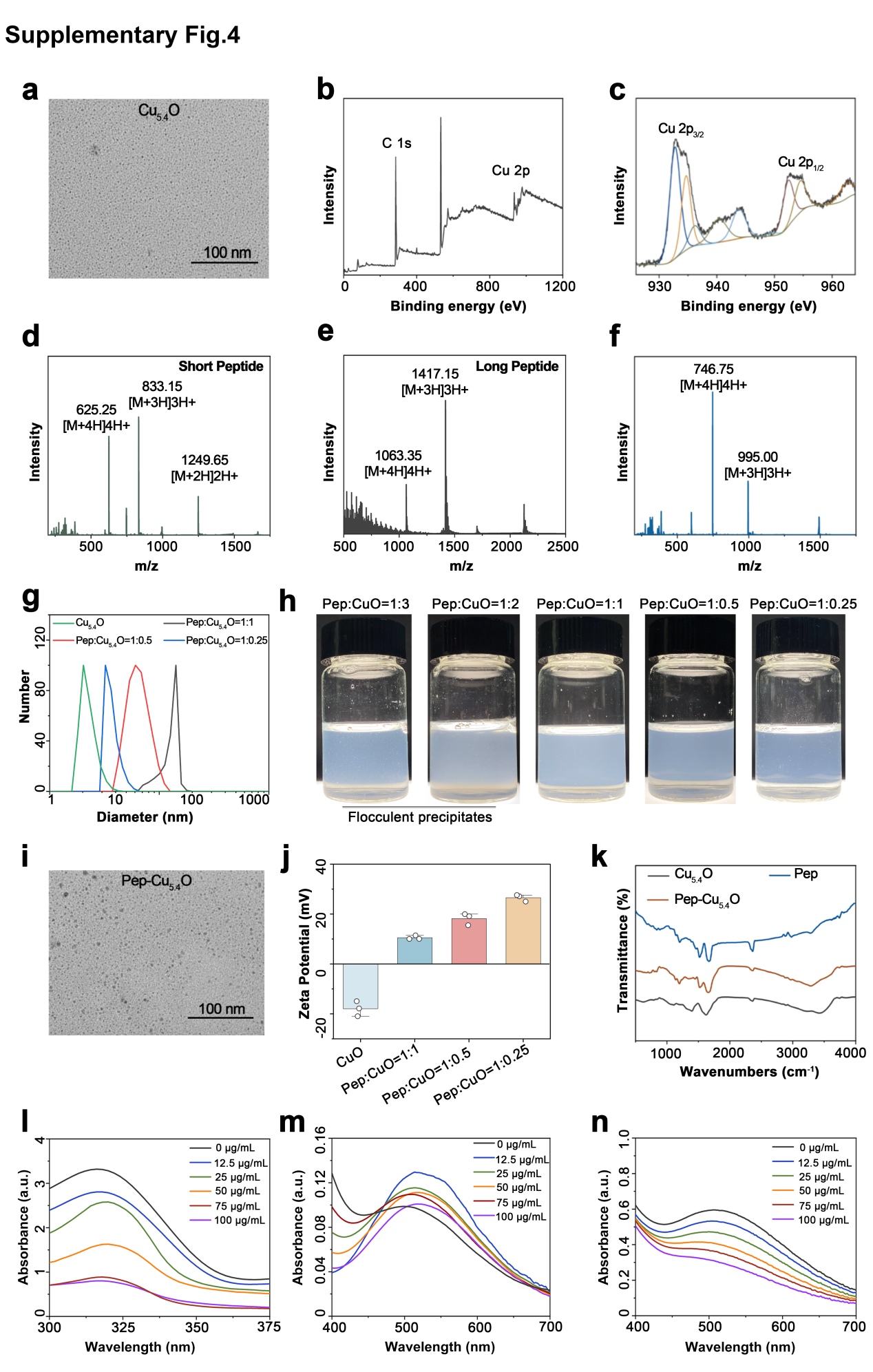
**

**Figure S4. (a)**TEM image of Cu_5.4_O. Scale bar: 100 nm. **(b, c)** XPS analysis of Cu_5.4_O XPS spectra **(b)** and Cu 2p XPS spectra of Cu_5.4_O **(c)**. **(d, e)** LC-MS analysis of peptide. LC-MS spectrum of short peptide **(d)** and long peptide **(e)**. **(f)** MMP-9-mediated proteolytic processing of Long Peptide characterized by LC-MS. **(g)** DLS of the chelation ratios of different peptides with Cu_5.4_O USNPS. **(h)** Visual assessment of chelating ratios between peptides and Cu_5.4_O USNPs. Macroscopically visible flocculent precipitates formed in solutions at chelating ratios of 1:3 and 1:2, indicating effective chelation. **(i)** TEM image of the Pep-Cu_5.4_O USNPS. Scale bar: 100 nm. **(j)** Zeta potential of the chelation ratios of different peptides with Cu_5.4_O. **(k)** FTIR spectra of Cu_5.4_O, Pep and Pep-Cu_5.4_O. **(l-n)** UV absorption diagram of the ROS scavenging efficiency of Pep-Cu_5.4_O@H151. ·OH **(l)**; H_2_O_2_ **(m)**; ·O_2_^-^ **(n)**.


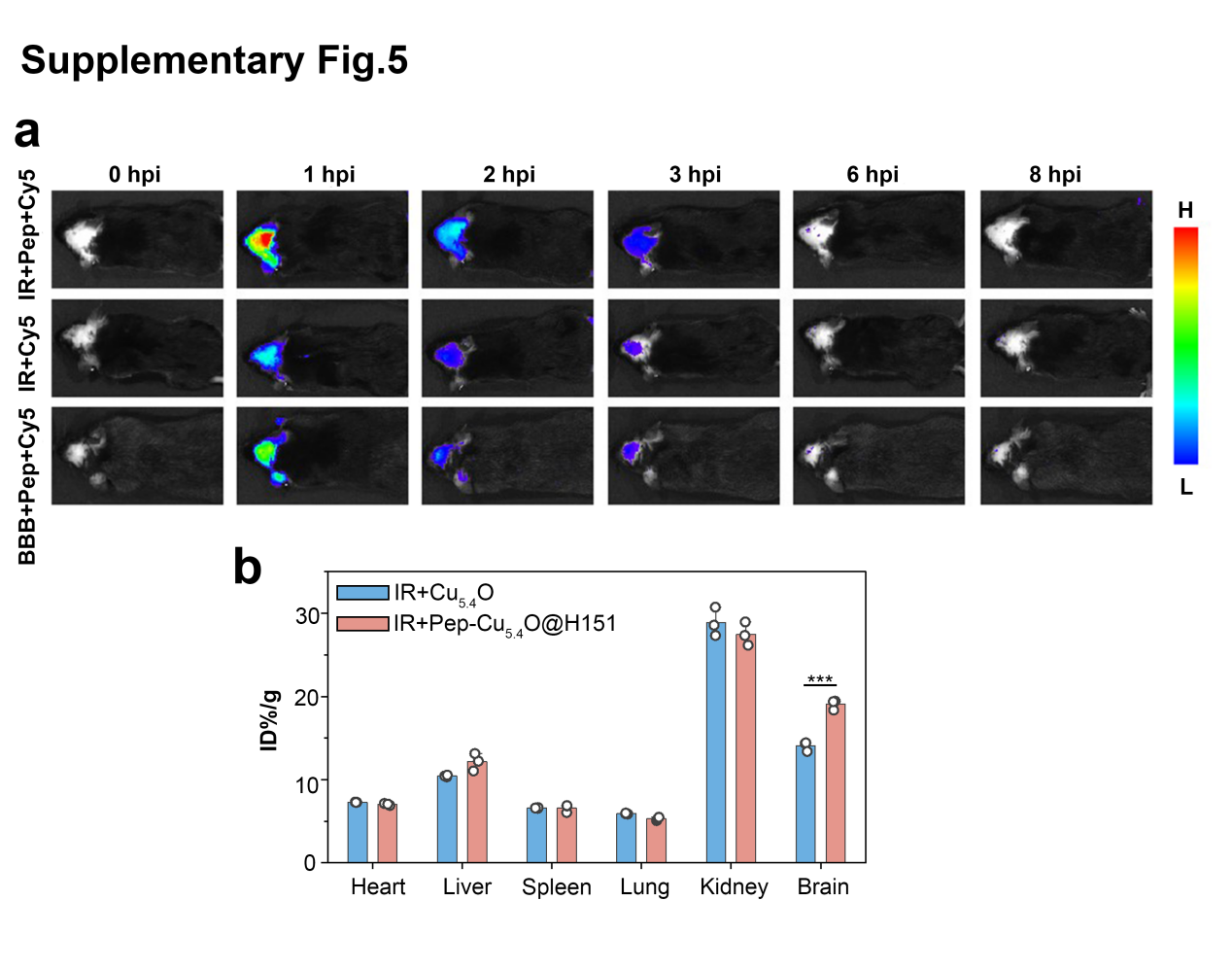


**Figure S5. (a)** Biodistribution analysis of a fluorescent tracer in mice using whole-body imaging. *In vivo* fluorescence imaging at 1-, 2-, 3-, 6-, and 8 hours post-intravenous injection (hpi) of a near-infrared fluorescent probe. **(b)** Biodistribution of Cu_5.4_O USNPs in major organs at 1 hour post-injection analyzed by inductively coupled plasma mass spectrometry (ICP-MS). All the statistical data are presented as the means ± SEMs.


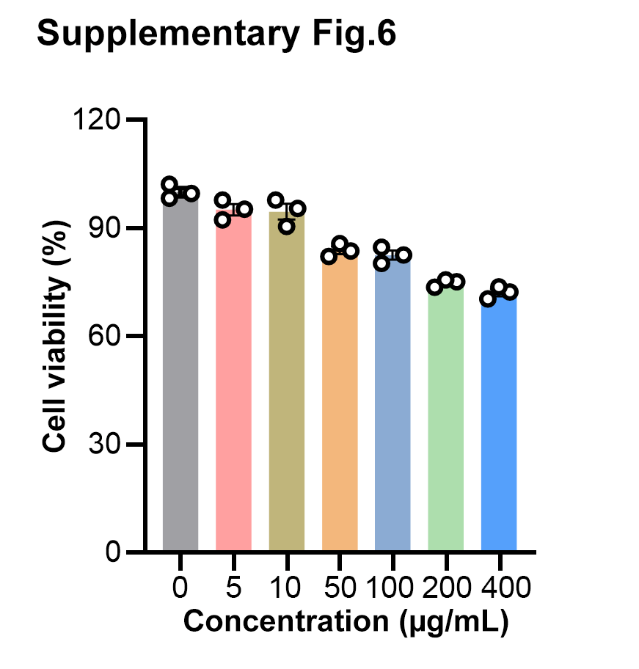


**Figure S6.** Cell viability of BV2 microglia treated with increasing concentrations of Pep-Cu_5.4_O@H151. Cytotoxicity was evaluated via CCK-8 assay 24 hours post-treatment.


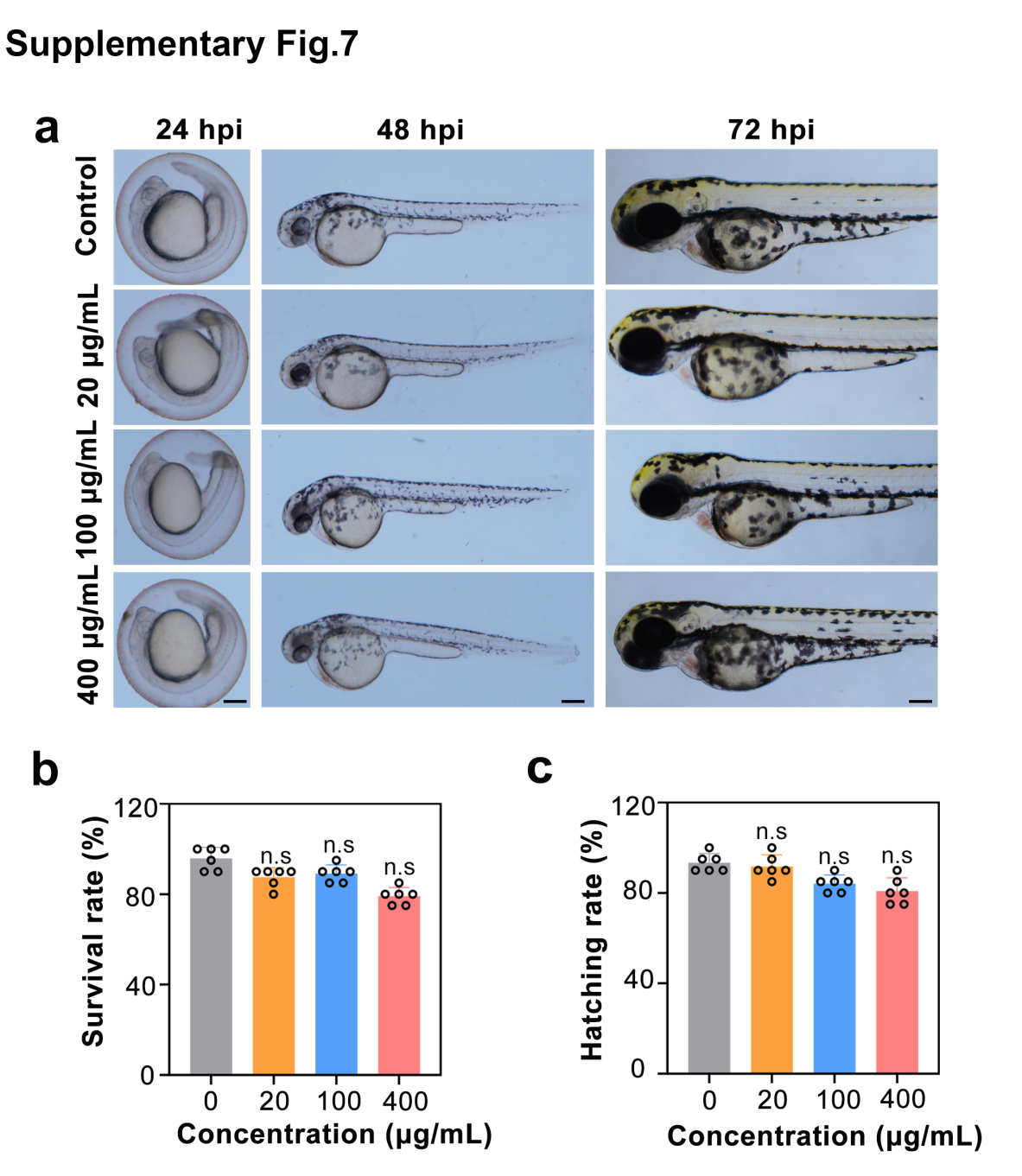


**Figure S7. (a)** Phenotypic analysis of zebrafish embryos at 24, 48, and 72 hpf when exposed to escalating concentrations of Pep-Cu_5.4_O@H151 (20, 100, 400 μg/mL). **(b)** Survival rates quantified at 24 hpf. **(c)** Hatching rates evaluated at 72 hpf (n = 20 embryos/group across six biological replicates). All the statistical data are presented as the means ± SEMs. Scale bars: 100 μm (a, 24 hpi and 72 hpi), 400 μm (a, 48 hpi).

**
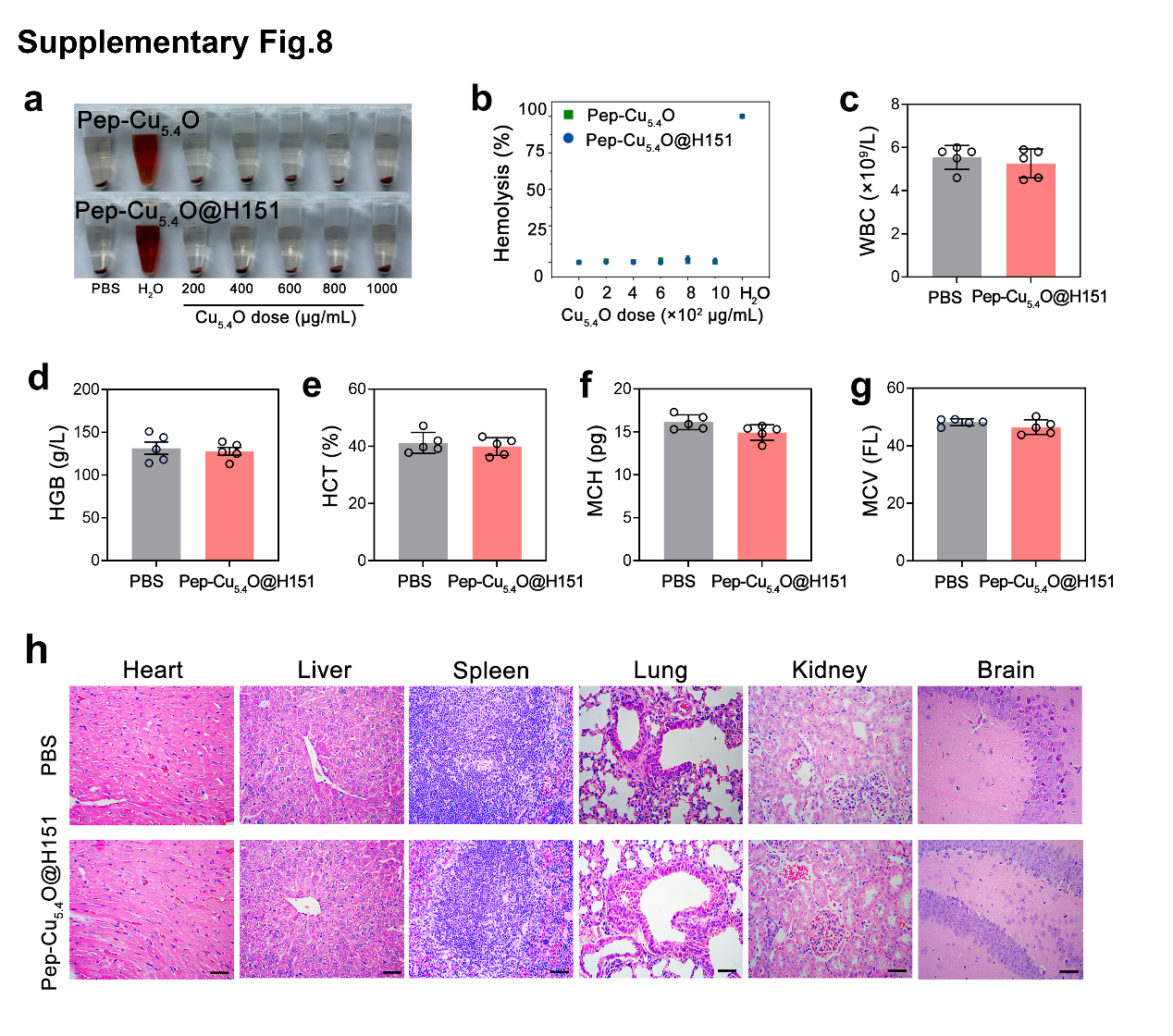
Figure S8. (a)** Representative images of hemolysis assays in mice erythrocytes treated with nanomaterials at concentrations of 200, 400, 600, 800, 1000 μg/mL. **(b)** Hemolytic ratio quantification (n = 5 mice). **(c-g)** Hematological parameters (WBC: white blood cells; HGB: hemoglobin; HCT: hematocrit; MCH: mean corpuscular hemoglobin; MCV: mean corpuscular volume) analysis (n = 5 mice; Student's *t*-test). **(h)** Histopathological evaluation via HE staining of major organs. heart, liver, spleen, lung, kidney and brain from mice treated with PBS or Pep-Cu_5.4_O@H151. All the statistical data are presented as the means ± SEMs. Scale bars: 50 μm.


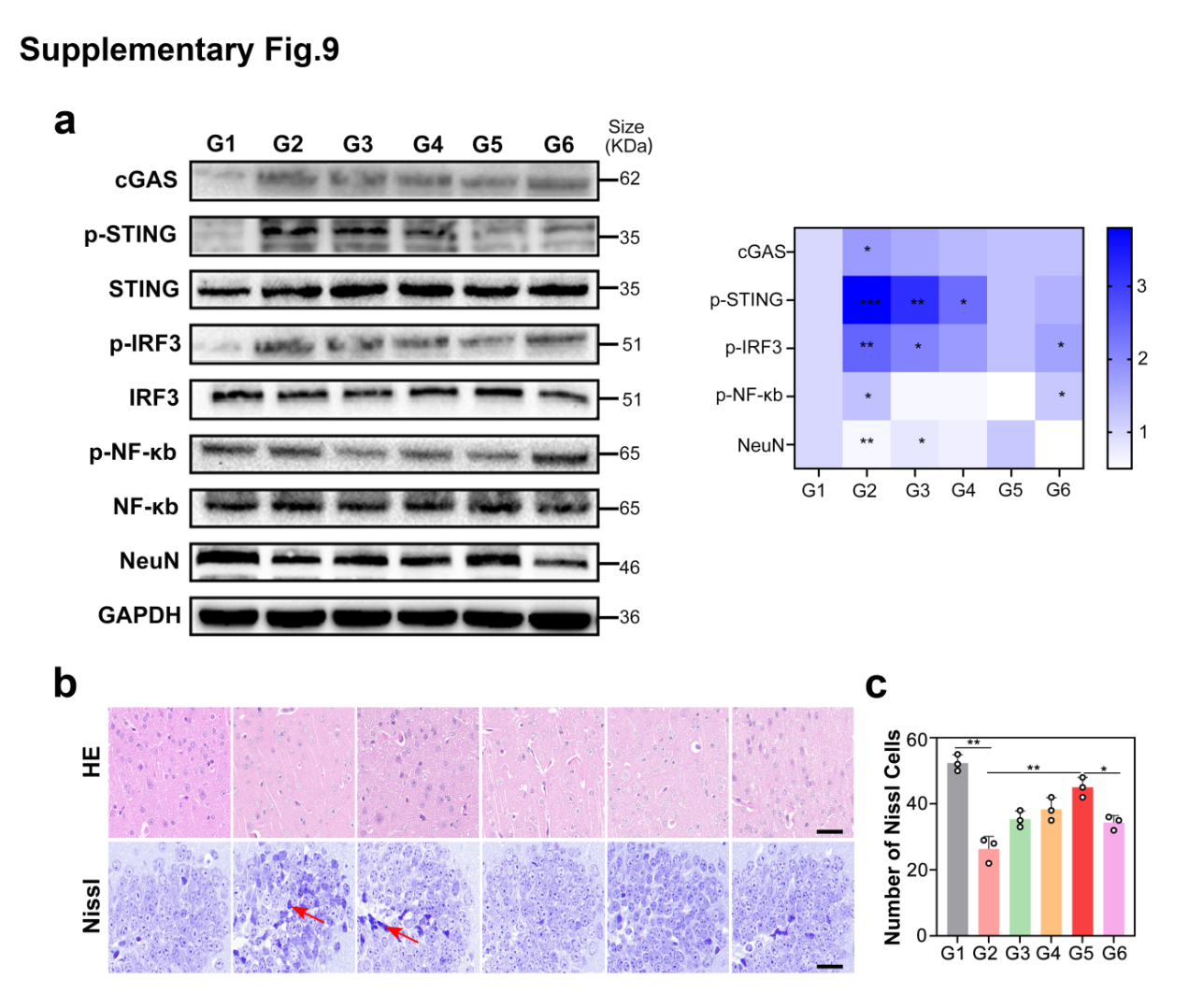


**Figure S9. (a)** Western blot analysis of protein expression in mice brain tissues. Representative Western blot images (left panel) depict protein bands of target molecules and the housekeeping protein GAPDH. Right panel shows quantitative data of target proteins, expressed as the ratio of target band intensity to GAPDH. n = 3 biological replicates; ***P<0.001, **P<0.01, *P<0.05; **Student’s *t*-test**. **(b)** H&E staining images of the prefrontal cortex and Nissl staining of the hippocampal dentate gyrus (DG). **(c)** Quantification of Nissl bodies per mm^2^ (n = 3 sections/group; one-way ANOVA test). All the statistical data are presented as the means ± SEMs. Scale bar: 20 μm.

**Supplementary Table 1.** The source of antibody

| **Antibody** | **Source** | **Identifier** |
| --- | --- | --- |
| cGAS | Cell Signaling Technology | #15102 |
| STING | Cell Signaling Technology | #13647 |
| p-STING | Cell Signaling Technology | #19781 |
| NF-κb | Cell Signaling Technology | #8242 |
| p-NF-κb | Cell Signaling Technology | #3033 |
| IRF3 | Cell Signaling Technology | #11904 |
| p-IRF3 | Cell Signaling Technology | #37829 |
| iNOS | Proteintech | #18985 |
| NeuN | Proteintech | #26975 |
| CD86 | Proteintech | #13395 |
| CD206 | Proteintech | #18704 |
| iba1 | GeneTex | #635363 |
| GFAP | Proteintech | #16825 |
| TNF-α | Santa Cruz Biotechnologies | #C2613 |
| γH2AX | Cell Signaling Technology | #9718 |
| p-Tau | Cell Signaling Technology | #15013 |
| β-Amyloid | Cell Signaling Technology | #9888 |
| GAPDH | Proteintech | #60004 |

**Supplementary Table 2.** List of primers used in this paper.

| Gene | Primer | Sequence (5′-3′) |
| --- | --- | --- |
| *mbp* | forward | AAGGGAAAGAGACCCCACCA |
|  | reverse | ATCGGCTTTCTCCAAAGCTCC |
| *shha* | forward | CGCCGTAATAGAGGACCAGG |
|  | reverse | TGGACCCCCTCCTGTTGTAA |
| *gad1b* | forward | TGGACCTGCAGAAGATCCTG |
|  | reverse | CTTCAGCAGGTTCAGCAACG |
| *ghrh* | forward | GCTGGAGCTGTTCAACCTCT |
|  | reverse | AGCCACAGAGAGGGTACAGG |
| *isl1* | forward | CAGCAAGACCCCAAAGACAC |
|  | reverse | TGGCGATCTTGTTGTTGTCC |
| *igf1* | forward | CCTGCTGCCTCTGGTCTATT |
|  | reverse | CTGGAAGCACACGGTCTCTC |
| *esr2b* | forward | GACGGCAACTACATCGACCT |
|  | reverse | TGGTGTTGCTGTTGATGAGG |
| *cyp19b* | forward | CTGCTGAAGGTGATGGAAGA |
|  | reverse | TTGTTGCCATTGTCTTCCTC |
| *trβ* | forward | TGCTGGAGAAGGTGCTGAAT |
|  | reverse | GGTGTGGTAGAGGTGGCTTC |
| *crhbp* | forward | CACCACCAACACCATCACCT |
|  | reverse | CAGGTCCAGGTCTTCACCAC |
| *crhr2* | forward | TGCCTTCCTCATCCTCCTCT |
|  | reverse | GGCAGGTAGAGGCTGTTGAG |
| Il-6 | forward | TAGTCCTTCCTACCCCAATTTCC |
|  | reverse | TTGGTCCTTAGCCACTCCTTC |
| Tnf-α | forward | CAGGGCAATCAACAAGA |
|  | reverse | CCTGGTCCTGGTCATCT |
| Il-1β | forward | TGCGGGCAATATGAAGTCA |
|  | reverse | TTCGCCATGAGCATGTCC |
| Ifn-γ | forward | CAGCAACAGCAAGGCGAAAA |
|  | reverse | TCAAGTGGCATAGATGTGGAAGAA |
| ND1 | forward | CCCTACCCACATCATTACCG |
|  | reverse | GAGCGTAAGTTGCGTGTTGT |
| Cox1 | forward | GCCCCAGATATAGCATTCCC |
|  | reverse | GTTCATCCTGTTCCTGCTCC |
| 18s rRNA | forward | CGGCTACCACATCCAAGGAA |
|  | reverse | GCTGGAATTACCGCGGCT |
| *β-actin* | forward | TTCACCACCACAGCCGAAAGA |
|  | reverse | TACCGCAAGATTCCATACCCA |

**Supplementary Movie 1 to 2**. Dynamic video of viable BV2 cells after irradiation, with mitochondria labeled in red (stained by PK Mito Deep Red) and dsDNA labeled in green (stained by SYBR).
